# Multilayer surface coating for enhanced anti-inflammation, anti-restenosis, and re-endothelialization in advanced biodegradable vascular stents

**DOI:** 10.1016/j.mtbio.2025.102570

**Published:** 2025-11-21

**Authors:** Duck Hyun Song, Seungwoon Baik, Jun Yong Kim, Jeong min Park, Byeongseok Ryu, Il Ho Seo, Su Sam Lee, Han Byul Kim, Young Joon Hong, Won-Gun Koh, WonHyoung Ryu, Yeu-Chun Kim, Dong Ryul Lee, Dong Keun Han

**Affiliations:** a Department of Biomedical Science, CHA University, South Korea; b ORANDBIO Co., Ltd., South Korea; c Department of Chemical and Biomolecular Engineering, Yonsei University, South Korea; d Department of Mechanical Engineering, Yonsei University, South Korea; e Department of Chemical & Biomolecular Engineering, Korea Advanced Institute of Science and Technology, South Korea; f Division of Radiation Biomedical Research, Korea Institute of Radiological and Medical Science, Seoul, South Korea; g Division of Cardiology, Chonnam National University Hospital, South Korea

**Keywords:** Coronary artery disease (CAD), Controlled drug release, Restenosis prevention, Spatiotemporal drug delivery, Endothelial regeneration, Anti-inflammation

## Abstract

Coronary artery disease (CAD) remains a leading cause of mortality worldwide, while conventional drug-eluting stents (DES) face limitations in long-term safety and controlled drug release. Here, we developed a poly (L-lactic acid) (PLLA)-based biodegradable vascular scaffold (BVS) with a three-layer coating using distinct technologies for spatiotemporal drug delivery. The luminal surface was selectively coated via electrospraying with alpha-lipoic acid (ALA) to promote endothelialization. The abluminal surface was treated using ultrasonic spray coating with magnesium hydroxide (MH) and everolimus (EVL) to suppress smooth muscle cell proliferation and inflammation. Finally, a precision dot-printing method was employed to deliver doxorubicin (DOX) for early inhibition of restenosis. This strategy enabled time- and site-specific drug release, achieving antioxidant, anti-inflammatory, and *anti*-restenotic effects while facilitating re-endothelialization. *In vivo* studies in a porcine model confirmed that the BVS-AEMD significantly reduced thrombosis and inflammation, underscoring its potential as a next-generation cardiovascular therapeutic platform.

## Graphical abstract

**Figure:**
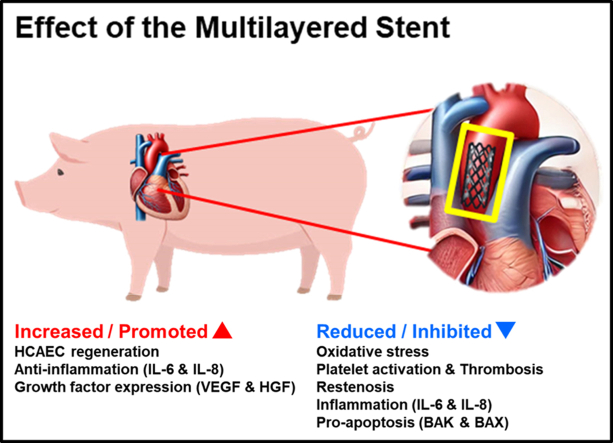

## 1 Introduction

Cardiovascular disease (CVD) remains the leading cause of death worldwide, accounting for over 17.9 million deaths each year [1]. It arises from lipid accumulation and endothelial dysfunction, which drive progressive atherosclerosis, narrowing the coronary arteries and restricting oxygen delivery to the myocardium [2]. Stent implantation has therefore become an essential clinical intervention to restore blood flow and prevent ischemic complications [3].

Since the introduction of bare-metal stents (BMS) in 1977, device technology has advanced to drug-eluting stents (DES) and, more recently, biodegradable vascular scaffolds (BVS) [4]. While DES significantly reduced restenosis through controlled antiproliferative drug release, both BMS and DES suffer from delayed endothelialization, late thrombosis, and chronic inflammation due to their permanent metallic structure [5]. BVS were designed to overcome these limitations via complete biodegradation and vascular restoration, but their thick struts and acidic degradation byproducts can still trigger inflammation and thrombosis [6].

Traditional coating strategies have applied drugs or inorganic particles uniformly to BVS surfaces without distinguishing between endothelial and smooth muscle cell layers [7,8]. This nonselective approach suppresses smooth muscle proliferation but simultaneously impairs endothelial recovery, delaying re-endothelialization and promoting restenosis [[9], [10], [11]]. Thus, a layer-specific strategy that independently supports endothelial healing while inhibiting smooth muscle overgrowth is essential.

Recent studies have sought to improve stent performance through spatially selective drug delivery and biofunctional coatings. Additive-manufactured and bioresorbable scaffolds have enhanced mechanical and degradation properties, but most designs still lack precise control over layer-specific localization and time-dependent release. Similarly, layer-by-layer coatings improve drug loading and stability but rely on passive diffusion rather than dynamic spatiotemporal coordination. Therefore, next-generation scaffolds must integrate both spatial selectivity and temporal control to achieve synergistic vascular regulation.

To address these challenges, we developed a multilayer-coated biodegradable vascular scaffold capable of spatially separated and temporally coordinated drug release. The base polymer, poly-L-lactic acid (PLLA), undergoes hydrolytic degradation into pyruvic acid and ultimately water and carbon dioxide. However, excessive acidic intermediates can lower local pH, leading to inflammation and impaired endothelial repair [12]. To counteract this, the luminal surface was functionalized with α-lipoic acid (ALA), a natural disulfide compound with potent antioxidant and anti-inflammatory properties [6,13,14]. ALA enhances nitric oxide bioavailability and reduces oxidative stress, thereby accelerating early endothelial regeneration and vascular stabilization [15,16].

The abluminal surface was engineered with magnesium hydroxide (MH) to neutralize acidic degradation products and modulate vascular tone, thrombosis, and smooth muscle proliferation [14]. MH also exhibits antithrombotic effects by reducing P-selectin expression, inhibiting fibrinogen–platelet complex formation, and lowering thromboxane A_2_ levels [14,15,17]. In parallel, everolimus (EVL) a rapamycin derivative with proven *anti*-restenotic efficacy was incorporated into the abluminal coating. Its spatial confinement minimizes interference with endothelial recovery while maintaining selective inhibition of smooth muscle proliferation and migration [12,18].

Previous single-layer coatings often resulted in uncontrolled burst release and insufficient temporal regulation. In contrast, the present multilayer configuration separates luminal and abluminal functions, achieving both cell-type specificity and balanced sustained release. MH provides long-term buffering and anti-inflammatory stability, while ALA and EVL act sequentially to promote early endothelialization and suppress mid-term hyperplasia.

To further refine release precision, electrohydrodynamic (EHD) micro-patterning was applied. Using a ring-electrode nozzle, drug-loaded PEG-DA ink was precisely deposited as uniform 10 μm dot arrays, followed by a PLGA over-coating to enhance drug encapsulation and sustain release. Unlike conventional dip or spray coatings that cause uneven films and burst kinetics, EHD printing provides high spatial accuracy and reproducible control [19]. This approach enables synchronized, predictable release across layers, ensuring both positional precision and kinetic stability.

In the outermost PEG-DA/PLGA matrix, doxorubicin (DOX) was incorporated at low, localized doses. Although DOX is known for cardiotoxicity [[20], [21], [22]], sub-cytotoxic concentrations can beneficially modulate oxidative stress and apoptosis, stabilizing the vascular wall and limiting smooth muscle proliferation [17]. Spatial confinement within the abluminal layer minimizes systemic exposure and off-target effects, contributing to safe and localized vascular healing [23].

Together, this multilayer configuration establishes a stage-specific therapeutic system. The ALA luminal layer delivers a rapid antioxidant burst that scavenges reactive oxygen species and promotes early endothelialization [13,14]. The EVL layer provides sustained antiproliferative activity to prevent smooth muscle overgrowth and mid-term restenosis [12,18]. The MH layer ensures long-term buffering and antithrombotic protection by neutralizing acidic degradation byproducts and reducing late inflammation [14,15,17].

This spatially and temporally responsive architecture represents a significant advancement over conventional uniform coatings. By integrating polymer degradation chemistry, pH regulation, and cell-type-specific drug release, the scaffold achieves synchronized healing enhancing endothelial recovery, suppressing smooth muscle proliferation, and preventing chronic inflammation [6,18,[24], [25], [26]]. The coordinated actions of ALA, EVL, and MH create a continuous transition from early vascular protection to long-term remodeling, offering a comprehensive solution to oxidative stress, restenosis, and degradation-associated inflammation in current BVS technology.

## 2 Materials and methods

### 2.1 Materials

The biodegradable vascular scaffold (BVS) used in this study was fabricated from poly (L-lactic acid) (PLLA) supplied by CGBio (Korea). It is a tube-shaped, laser-cut PLLA scaffold with an average strut thickness of approximately 150 μm and an internal diameter of 3.0 mm. Poly (D,L-lactide) (PDLLA; Resomer® R 205 S) and Poly (D,L-lactide-co-glycolide) (PLGA, RG 505, Mw = 24 kDa) was obtained from Evonik Industries AG (Germany). Alpha-lipoic acid (ALA), 1-ethyl-3-diaminopropyl carbodiimide (EDC), N-hydroxysuccinimide (NHS), rhodamine B, magnesium hydroxide (MH), stannous octoate, albumin from human serum, L-lactate dehydrogenase (LDH), and fibrinogen from human plasma were obtained from Sigma-Aldrich (USA). Tetrahydrofuran (THF) and hydrogen peroxide were secured from Duksan Pure Chemicals (Korea), while trypsin/ethylenediaminetetraacetic acid (trypsin/EDTA), acetoxymethyl calcein (calcein-AM), and ethidium homodimer-1 (EthD-1) were procured from Thermo Fisher Scientific (USA).

Everolimus (EVL) was acquired from CGBio (Korea), and poly (ethylene glycol) (PEG5K, MW = 5000) was sourced from Laysan Bio (USA). Chloroform (CF), dichloromethane (DCM), and nitric acid were provided by Daejung Co., Ltd. (Korea). The universal RNA extraction kit was sourced from Bioneer (Korea). Platelets were obtained from the blood center of the Korean National Red Cross (Korea). The 20 % SDS solution was secured from Bio-Rad (USA).

Human coronary artery endothelial cells (HCAECs), endothelial cell growth basal medium-2 (EBM-2), the EGM-2 MV BulletKit, human coronary artery smooth muscle cells (HCASMCs), smooth muscle cell growth basal medium, and the SmGM BulletKit were purchased from Lonza (Switzerland).

Dulbecco's phosphate-buffered saline (DPBS) was acquired from WELGENE (Korea), and the cell-counting kit (CCK-8) was obtained from Dongin LS (Korea). The nuclease-free water and lipophilic trace 3,3′-dioctadecyloxacarbocyanine perchlorate (DiO) were procured from Invitrogen (USA), and 2′,7′-dichlorodihydrofluorescein (DCF-DA) was sourced from Cayman Chemical (USA). All chemicals were laboratory reagent grade and used without purification. Yorkshire × Landrace F1 crossbred castrated male pigs were supplied 5–10 days prior to the experiment by the laboratory animal center of Chonnam National University Medical Institute.

### 2.2 Preparation and characterization of the BVS-A

To achieve sustained drug release from the BVS in aqueous conditions, the BVS was coated with an antioxidant drug ALA using the electrospraying method. Biodegradable PDLLA served as the drug carrier, while ALA was selected as the antioxidant drug due to its ability to reduce oxidative stress at the implantation site. An ALA-containing PDLLA solution was prepared by dissolving a mixture of 0.3 wt% PDLLA and 0.015 wt% (72.57 μM) ALA in THF under ambient conditions, stirred for 24 h while protected from light. This solution was then transferred to a 12 mL syringe equipped with a 25G metal needle. The electrospraying conditions were set with a flow rate of 1.5 mL/h, humidity maintained between 65 % and 70 % at room temperature, a needle-to-collector distance of 15 cm, and an applied voltage of 10 kV. In addition, to achieve spatially selective coating, the fusion scaffold was covered with a conductive aluminum material to block deposition on the outer surface, while the electric field was directed toward the inner luminal side of the cylindrical mesh structure, ensuring that the electrosprayed coating was predominantly formed on the inner surface. The BVS was vertically fixed in a custom-made holder and sprayed for 5 min. The ALA-coated BVS (BVS-A) was rinsed with deionized water and PBS solution to remove any residual solvent. Surface morphology was characterized using a field emission-scanning electron microscope (FE-SEM) (JEOL-7610 F-Plus, JEOL Co., Japan), and further confirmed for film samples by atomic force microscopy (AFM, DektakXT Stylus Profiler, Bruker, USA). To visualize the electrosprayed coating, rhodamine B was used instead of ALA in the PDLLA solution, and fluorescence images were captured with a fluorescent microscope (Olympus Corporation, Japan). The drug release profile was determined using a UV/Vis NanoDrop spectrophotometer (Thermo Fisher Scientific, MA, USA). The BVS-A was immersed in PBS solution and incubated at 37 °C to facilitate ALA release from the electrosprayed PDLLA solution. Periodically, 2 μL samples of the solution were taken, and absorbance at 330 nm was measured to monitor the release.

### 2.3 Synthesis of ODLLA-grafted magnesium hydroxide (GF-MH)

ODLLA-grafted magnesium hydroxide (GF-MH) was synthesized via ring-opening polymerization of D,L-lactide in the presence of MH. Briefly, 4 g of MH and 8 g of D,L-lactide were finely ground using a mortar and pestle to obtain a homogeneous powder mixture. Stannous octoate was added as a catalyst at a molar ratio of 1:10,000 relative to D,L-lactide, along with a small volume of toluene to ensure uniform dispersion due to the high viscosity of the catalyst.

The mixture was transferred into a glass ampoule containing a magnetic stir bar. The ampoule was first preheated at 220 °C for 1 min, then evacuated and sealed. The sealed ampoule was subsequently maintained at 150 °C for 16 h in a silicone oil bath under vacuum.

After completion, the ampoule was rapidly quenched in liquid nitrogen to solidify the contents. The glass was broken to recover the solid product, which was then immersed in a chloroform:acetone (1:1, v/v) mixture to remove unreacted monomers and residual oligomers. The mixture was further ground for 30 min using a mortar and pestle and subjected to centrifugation at 5000 rpm for 10 min at 4 °C. This washing step was repeated three times. Finally, the purified GF-MH particles were vacuum-dried at 25 °C for 24 h prior to use.

The chemical structure of the obtained GF-MH particles was confirmed by Fourier transform infrared spectroscopy (FTIR, PerkinElmer, USA). Thermal stability was evaluated using thermogravimetric analysis (TGA, PerkinElmer, USA). The particle size distribution was determined by dynamic light scattering (DLS, Malvern, UK), and the neutralization effect of GF-MH was verified by monitoring the pH of the aqueous suspension.

### 2.4 Preparation and characterization of the BVS-AEM

In previous studies, due to the difficulty of uniform dispersion of hydrophilic surface modifiers on stents, a hydrophilic to hydrophobic transformation was applied to disperse magnesium hydroxide (MH) within PLLA matrices. All synthesized MH samples were ground using a wet grinder from NETZSCH at 3000 rpm with 0.1 mm grinding beads, passing through a 0.6 mesh screen for 90 min. BVS was coated using an ultrasonic spray coater (Noanix, Korea). The coating solution was prepared by dissolving 3.7 mg/mL EVL with 0.5 wt% PDLLA and further dispersing 0.15 % nano-magnesium hydroxide (GF-MH) in THF to obtain BVS-E and BVS-AEM formulations. The prepared solution was then applied onto the surface of BVS-A scaffolds. In addition, the ultrasonic spray coating process was performed under the following optimized conditions: nozzle setpoint of 2 W, coating duration of 10 min (2 cycles), jog velocity of 0.03 cm/s, pump flow rate of 0.05 mL/min, focus gas flow of 0.4 L/min, and rotation speed of 150 rpm.

Surface morphology and element distribution of the BVS-AEM were visualized via FE-SEM. To observe the surface roughness of the coating, atomic force microscopy (AFM) was employed, which was measured using film samples. The presence of coated GF-MH and EVL was confirmed using FTIR. The loading amount of EVL was measured by HPLC with a mobile phase of water, acetonitrile, and methanol at a flow rate of 1.00 mL/min, quantifying EVL emission at 278 nm over 10 min. The loading amount of GF-MH was determined using an inductively coupled plasma optical emission spectrometer (ICP-OES; Optima 8000, PerkinElmer, MA, USA) after dissolving each BVS in a 5 % nitric acid solution.

### 2.5 Preparation and characterization of the BVS-AEMD

The MSN-SS-PEG nanoparticles were synthesized through a thiol–disulfide exchange reaction, followed by PEGylation to enhance colloidal stability and biocompatibility. To achieve reproducible and uniform multilayer coatings, both the electrohydrodynamic (EHD) dot-printing parameters and MSN-SS-PEG synthesis conditions were systematically optimized. The optimization aimed to ensure stable nanoparticle dispersion, uniform drug deposition, and controlled release kinetics under physiological conditions. Detailed optimization parameters and rationale are provided in Fig. S1.

### 2.6 Blood compatibility of the BVS-AEMD

The PLLA film, as the base material of the BVS, was sterilized under UV light for 10 min and then hydrated in 1 mL of PBS solution for 1 h. For protein adsorption analysis, the scaffolds were incubated in 0.2 mg/mL fibrinogen solution or 3 mg/mL albumin solution at 37 °C for 1 h in the presence of 10 μM GSH or 10 μM SNAP, respectively. After incubation, the samples were washed three times with distilled water and immersed in 5 % SDS solution at 37 °C overnight. The concentration of eluted proteins was measured using a micro-BCA kit.

A platelet solution with a concentration of 5 × 10^4^ platelets/μL was prepared from platelet-rich plasma (PRP) and platelet-poor plasma (PPP). The BVS samples were immersed in 1 mL of the platelet solution and incubated at 37 °C for 2 h. After incubation, the samples were rinsed three times with PBS solution. For SEM imaging of adherent platelets, the samples were fixed in 2.5 % glutaraldehyde solution for 1 h and then sequentially dehydrated in ethanol solutions of 50 %, 60 %, 70 %, 80 %, 90 %, and 100 %. To quantify the adhered platelets, a 2 % Triton X-100 solution was added to lyse the platelets for 15 min, and LDH activity in the lysates was assessed using an LDH assay kit according to the manufacturer's instructions.

### 2.7 Evaluation of cell culture, migration, and proliferation

HCAECs and HCASMCs, each at passage less than 7, were cultured in T75 tissue culture flasks with 13 mL of EGM-2 or SmGM, respectively, in a humidified environment with 5 % CO_2_ at 37 °C. HCAECs and HCASMCs were then seeded at 1 × 10^5^ cells/mL on each BVS. After 24 h, the medium was removed, and 400 μL of 10 % CCK-8 solution was added to each well in the dark. Following a 2 h incubation, absorbance was measured at 450 nm using a SpectraMax M2 plate reader (Molecular Devices, USA). For the scratch assay, HCAECs were seeded into 6-well plates at a density of 1.5 × 10^5^ cells per well. Upon reaching 100 % confluence, a sterile 1 mL pipette tip was used to scratch the monolayers. Each BVS was treated with an indirectly co-cultured system using trans-well inserts (SPLInsert™, SPL, Korea), and plates were incubated at 37 °C in 5 % CO_2_ for 9 h. Cell migration was measured using ImageJ software (ImageJ 1.44p, National Institutes of Health, Bethesda, USA).

### 2.8 Antioxidant and inflammatory activity

To assess the antioxidant activity of released ALA, cell culture media containing 300 μM hydrogen peroxide was prepared to induce oxidative stress in the cells. Sterilized ALA-BVS was placed in this media and incubated at 37 °C for 72 h to release ALA and reduce the reactive oxygen species present. This conditioned media was subsequently used for cell culture. Cell proliferation was quantitatively measured using a CCK-8 assay after 24 h, with a control group consisting of bare BVS treated and incubated under the same conditions. HCAECs were cultured in 24-well tissue culture plates and exposed to the conditioned media for one day at 37 °C. The cells were then incubated with 20 μM DCF-DA solution in media for 45 min at 37 °C in the dark. After being washed three times with PBS solution, live cells and DCF-DA positive cells were visualized using a fluorescence microscope (CKX53; Olympus, Japan).

### 2.9 RNA extraction and quantitative real-time PCR

RT-qPCR was performed to investigate the expression of genes related to anti-inflammation and angiogenesis *in vitro*. HCAECs and HCASMCs were cultured and treated with conditioned media for 24 h, following the same procedure described in the “Antioxidant assay." Total cellular RNA was extracted using the AccuPrep® Universal RNA Extraction Kit (Bioneer, Korea), according to the manufacturer's instructions. The extracted RNA was then used to synthesize cDNA with the PrimeScript RT Reagent Kit (Takara, Japan). RT-qPCR was conducted using SYBR Green PCR Master Mix (Applied Biosystems, USA) and specific primers. The expression of genes associated with angiogenic, and inflammatory processes were quantified using 18 S rRNA as a reference gene and the 2Ct method. Their sequences are as follows: 18 S rRNA (18 S: forward, 5′-gcaattattccccatgaacg-3′ and reverse, 5′-gggacttaatcaacgcaagc-3′); BCL2 Antagonist/Killer 1 (BAK: forward, 5′-agacctgaaaaatggcttcg-3′ and reverse, 5′-cggaaaacctcctctgtgtc-3′); BCL2-Associated X, Apoptosis Regulator (BAX: forward, 5′-catcatgggctggacattg-3′ and reverse, 5′-gggacatcagtcgcttcagt-3′); B-cell Lymphoma 2 (BCL-2: forward, 5′-agtacctgaaccggcacct-3′ and reverse, 5′-gccgtacagttccacaaagg-3′); Vascular Endothelial Growth Factor A (VEGF: forward, 5′-actggaccctggctttactg-3′ and reverse, 5′-tctgctccccttctgtcgt-3′); Hepatocyte Growth Factor (HGF: forward, 5′-cagcatgtcctcctgcatc-3′ and reverse, 5′-tcttttcctttgtccctctgc-3′); Interleukin-6 (IL-6: forward, 5′-gatgagtacaaaagtcctgatcca-3′ and reverse, 5′-ctgcagccactggttctgt-3′); Interleukin-8 (IL-8: forward, 5′-agacagcagagcacacaagc-3′ and reverse, 5′-atggttccttccggtggt-3′); Superoxide Dismutase 1 (SOD1: forward, 5′-gactgactgaaggcctgcat-3′ and reverse, 5′-acatcggccacaccatcttt-3′); Superoxide Dismutase 2 (SOD2: forward, 5′-aaacctcagccctaacggtg-3′ and reverse 5′-acatcaatccccagcagtgg-3′); Alpha-Smooth Muscle Actin (α-SMA: forward, 5′-gaagaggacagcactgcctt-3′ and reverse 5′-tcccagttggtgatgatgcc-3′); Rho-Associated Coiled-Coil Containing Protein Kinase 1 (ROCK1: forward, 5′-ggtggtcggttggggtattt-3′ and reverse 5′-ctggtgctacagtgtctcgg-3′); The data were quantified using 2-ΔΔCt method with 18 s rRNA as a reference.

### *2.10* *In vivo* surgical procedure for BVS implantation

The Ethics Committee of Chonnam National University Medical School and Chonnam National University Hospital approved this animal study (CNUHIACUC-21013), adhering to the Guide for the Care and Use of Laboratory Animals by the US National Institutes of Health (Publication No. 85–23, revised 1996). A total of 15 pigs (8 weeks old, 25–30 kg) were used in this study and were randomly assigned to either the control BVS group or the BVS-AEMD group (n = 7 and n = 8, respectively). Pigs were anesthetized with xylazine (3 mg/kg), tiletamine and zolazepam (2.5 mg/kg), and azaperone (6 mg/kg). An intravenous (IV) catheter was inserted into the marginal ear vein for the administration of emergency medications and fluids, such as epinephrine and antiarrhythmic agents (amiodarone hydrochloride). Continuous IV infusion of 0.9 % saline was maintained throughout the experiment. The pigs were intubated, and anesthesia was sustained with 1 % sevoflurane in oxygen. To alleviate pain, tramadol HCl (5 mg/kg) was administered intravenously both before and after the procedure. A coronary artery from an 8-week-old pig was used, with angiography performed to confirm stent placement. The left anterior descending and left circumflex arteries were used for the study. Additionally, the pigs were given 75 mg clopidogrel and 100 mg aspirin daily for five days prior to the procedure.

### 2.11 Optical coherence tomography analysis

For the study of neointima in pig blood vessels, the carotid artery was excised and examined using optical coherence tomography (OCT; Model C7Xr Dragonfly Optis Imaging Catheter, St. Jude Medical, MIN, USA). The coronary artery was then secured to a guide wire linked to a water box designed for *in vitro* experiments. An imaging catheter (C7 Dragonfly) was introduced into the coronary artery via the guide wire. OCT images were captured by connecting the Dragonfly Duo to the imaging catheter, and neointimal vessel measurements were performed using Light-Lab imaging software (offline review workstation).

### 2.12 Histopathological and immunohistochemical analyses

Harvested coronary artery tissues were fixed in 4 % paraformaldehyde (Wako, Japan), embedded in paraffin, and sectioned at a thickness of 5 μm. Immunohistochemical staining was performed with anti-alpha smooth muscle actin (α-SMA, 1:100; Abcam, Cambridge, UK) and anti-CD34 (ab108595, Abcam, USA) primary antibodies, followed by appropriate secondary antibodies. In addition, hematoxylin and eosin (H&E) and Carstair's staining were carried out to evaluate tissue morphology and histological characteristics.

### 2.13 Statistical analysis

All statistical analyses were accomplished using GraphPad Prism (San Diego, CA, USA). One-way ANOVA with Tukey's multiple comparison posttest was performed to compare the samples (n ≥ 3). Results were not considered significant (ns) when *p* > 0.05 and statistically significant when ∗*p* < 0.05, ∗∗*p* < 0.01, and ∗∗∗*p* < 0.001, and ∗∗∗∗*p* < 0.0001.

## 3 Results and discussion

### 3.1 Characterization of luminal ALA-coated biodegradable vascular stent

To achieve sustained antioxidant delivery, poly (D,L-lactide) (PDLLA), which degrades more slowly than other biodegradable polymers, was selected as the carrier matrix for α-lipoic acid (ALA). The PDLLA–ALA coating was fabricated using an electrospraying technique optimized for viscosity and spray stability, based on a previously reported electrospinning method [27].

**Fig. 1:**
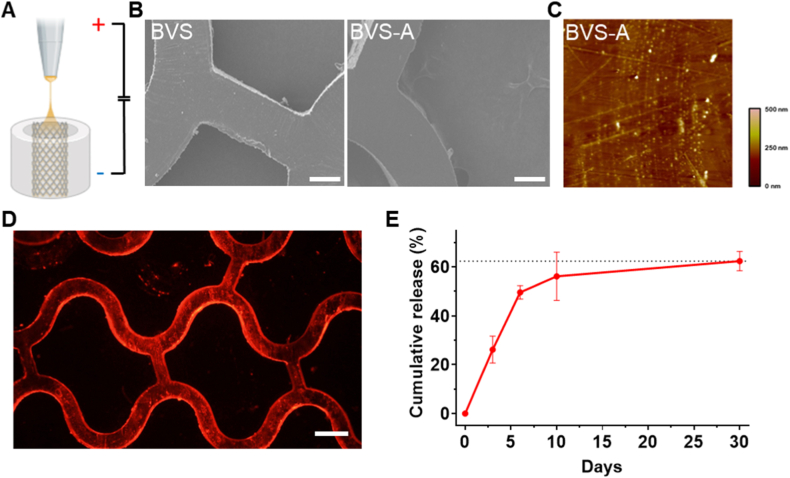
**Characterization of ALA-loaded BVS (BVS-A):** (A) Schematic illustration of luminal surface coating using conductive and ALA spray, (B) SEM images of bare BVS and ALA-loaded BVS (BVS-A) (Scale bar = 100 μm), (C) 3D-AFM microstructure of the ALA-modified PLLA (PLLA-A) surface, (D) Fluorescence images of Nile Red (NR)-labeled BVS-A-Rho coatings (Scale bar = 200 μm), and (E) Cumulative ALA release profile from BVS at 37 °C for 30 days.

A polymer solution containing ALA was loaded into a 12 mL syringe equipped with a 25-gauge metal needle positioned 15 cm from the collector, and a voltage of 10 kV was applied. The biodegradable vascular scaffold (BVS) was vertically fixed in a custom-designed holder and sprayed for 5 min to produce the ALA-coated BVS (ALA-BVS). This process enabled selective luminal coating of PDLLA-based particles, forming micro/nano-sized particles uniformly distributed on the PLLA substrate (Fig. 1A).

To determine optimal coating parameters, PDLLA concentrations of 0.1 %, 0.2 %, and 0.3 % were tested (Fig. S6). At 0.1 % and 0.2 %, the coating process adversely affected the outer wall, resulting in slight surface deformation, whereas 0.3 % PDLLA produced a smooth surface without altering the outer morphology. Coating durations of 2, 5, and 10 min were also evaluated, and a 5-min coating time produced the most uniform surface (Fig. S6). Since excessive surface roughness can promote thrombogenesis and reduce biocompatibility, a 0.3 % PDLLA concentration with a 5-min coating duration was selected as optimal for subsequent experiments [28].

SEM imaging confirmed that the coated samples maintained a smooth morphology comparable to the uncoated BVS (Fig. 1B). AFM analysis further verified that the coating process did not affect surface roughness (Fig. 1C); the average roughness (Rz) was 20.866 nm—well below the 2 μm threshold known to promote platelet adhesion [29] indicating minimal risk of thrombosis or inflammatory response. To verify coating uniformity, rhodamine B was incorporated into the coating solution, and fluorescence imaging revealed a homogeneous luminal fluorescence pattern, confirming uniform ALA deposition across the inner surface (Fig. 1D). These results demonstrate that the electrospraying process achieved a stable, reproducible, and luminally selective coating suitable for endothelialization-promoting drug delivery.

ATR-FTIR spectroscopy of BVS, ALA, and BVS-A samples was performed to analyze chemical bond changes (Fig. S2). The ALA spectrum displayed characteristic absorption bands at 2,927, 2,865, and 2844 cm^−1^, corresponding to asymmetric and symmetric stretching of C–H bonds, and a strong band at 1688 cm^−1^ attributed to carbonyl (C=O) stretching [30]. These peaks indicate the presence of aliphatic chains and carbonyl groups characteristic of ALA's molecular structure. The persistence of these peaks confirms that ALA retained its chemical integrity during the coating process and remained stably present on the stent surface [31].

HPLC analysis determined the ALA loading amount and drug-loading efficiency to be 50.6 μg and 30.7 %, respectively (Table S1). High loading efficiency ensures sufficient drug availability at the target site, thereby enhancing the stent's therapeutic efficacy [32]. The cumulative release profile of ALA followed first-order release kinetics (R^2^ = 0.9526), exhibiting an initial burst release of approximately 55 % within 10 days and a total release of 60 % by 30 days (Fig. 1E). The sustained 30-day release was attributed to the slow degradation rate of the PDLLA matrix.

Because ALA is hydrophilic, it typically exhibits rapid release within a few days; however, in this study, the gradual degradation characteristics of PDLLA enabled sustained ALA release for more than 30 days. This prolonged release provides continuous antioxidant effects not only during the early post-implantation phase but also throughout the intermediate stage of re-endothelialization, thereby reducing oxidative stress, supporting endothelial function, and suppressing smooth muscle proliferation [27].

Clinically, re-endothelialization should be completed within approximately one month after stent implantation—a critical period for restoring endothelial integrity and preventing thrombosis. Therefore, the ALA release profile was intentionally designed to match this one-month healing window.

In conventional coating techniques, selective luminal coating is technically challenging, as most processes result in both luminal and abluminal deposition or require complex masking jigs. Consequently, previous studies have primarily focused on abluminal coatings designed to inhibit smooth muscle proliferation, limiting the use of luminal coatings aimed at enhancing endothelial recovery. The electrospraying technique used in this study overcomes these limitations by enabling spatially confined deposition of PDLLA-ALA exclusively onto the luminal surface, thereby delivering antioxidant protection directly to the endothelium-facing interface where oxidative damage is most severe.

In summary, the optimized PDLLA-ALA luminal coating achieved a uniform, stable morphology while ensuring sustained ALA release for over 30 days. The coating preserved ALA's chemical stability, exhibited high drug-loading efficiency, and aligned with the clinically relevant one-month re-endothelialization period. These characteristics collectively demonstrate that the PDLLA-ALA coating serves as an effective luminal antioxidant layer capable of reducing oxidative stress, maintaining endothelial function, and promoting vascular healing following biodegradable scaffold implantation.

HPLC analysis determined the ALA loading amount and drug-loading efficiency to be 50.6 μg and 30.7 %, respectively (Table S1). High loading efficiency is essential for ensuring sufficient drug availability at the target site, thereby enhancing the stent's therapeutic efficacy [32]. The cumulative release profile of ALA followed first-order release kinetics (R^2^ = 0.9526), exhibiting an initial burst release of approximately 55 % within 10 days and a total release of 60 % by 30 days (Fig. 1E). The sustained 30 days release was attributed to the slow degradation rate of the PDLLA matrix.

### 3.2 Characterization of abluminal MH–EVL coating on biodegradable vascular stent

On the abluminal surface of the scaffold, a dual-functional coating was developed to overcome the limitations of conventional BVS. Uniform drug coatings typically fail to differentiate between endothelial and smooth-muscle layers, leading to delayed re-endothelialization, chronic inflammation, and restenosis. In addition, acidic byproducts generated during PLLA degradation create a hostile microenvironment that triggers platelet activation and smooth-muscle proliferation.

**Fig. 2:**
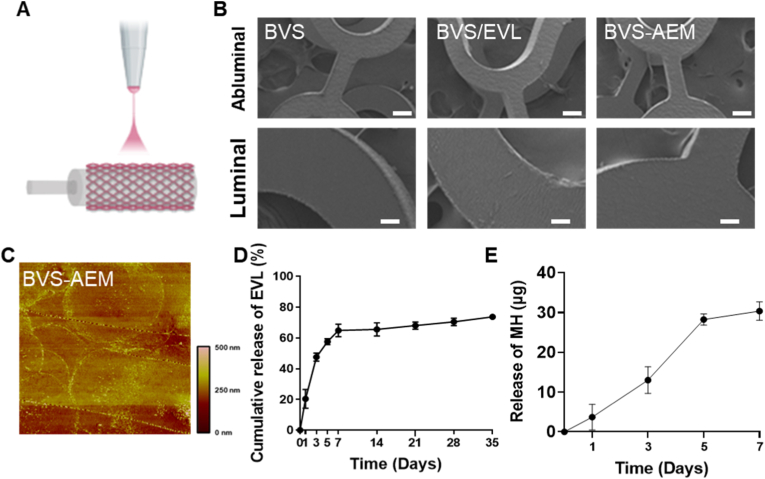
**Characterization of a Coated Support for pH Neutralization and Restenosis Prevention (BVS-AEM):** (A) Abluminal coating process using electrosprayed MH and EVL, (B) SEM images of BVS, BVS/EVL (Scale bar = 100 μm), and BVS/AEM coatings, (C) 3D-AFM surface microstructure of the AEM-modified PLLA (PLLA-AEM), (D) Cumulative release profile of EVL from BVS/AEM at 37 °C for 35 days, and (E) Cumulative release profile of MH from BVS/AEM at 37 °C for 7 days.

To counter these effects, the outer surface was engineered with a composite of pharmacological and inorganic components everolimus (EVL), an mTOR inhibitor that suppresses vascular smooth muscle cell (VSMC) proliferation, and magnesium hydroxide (MH), which neutralizes acidic degradation products (Fig. 2A).

As reported previously, MH was surface-modified with oligo (D,L-lactide) (ODLLA) to prevent aggregation and improve dispersion. FTIR confirmed successful ODLLA grafting through the ester carbonyl (–C=O) peak at 1709 cm^−1^ (Fig. S3A), and TGA indicated that ≈14 wt% of the total mass was grafted ODLLA (Fig. S3B). In organic solvents, unmodified MH aggregated at the micron scale, whereas MH-ODLLA maintained a stable particle size of ≈300 nm, demonstrating excellent dispersion (Fig. S3C). Importantly, MH-ODLLA retained pH-buffering capacity comparable to unmodified MH (Fig. S3D), confirming that surface modification improved colloidal stability without compromising buffering ability [33,34]. This modification enabled uniform dispersion of MH in the hydrophobic PLLA matrix, ensuring compatibility while preserving its neutralization capacity.

Using this approach, a composite abluminal coating composed of MH, EVL, and PDLLA was applied to the BVS to mitigate oxidative stress–induced inflammation, delayed endothelialization, and mechanical limitations of conventional stents. PLLA films were used as a model substrate for characterization. After coating, the surface remained smooth, and no significant differences in roughness were observed among BVS, BVS-E, and BVS-AEM groups (Fig. 2B). AFM analysis showed an average roughness (Rz) of 94.989 nm, indicating that the coating process did not alter surface topography (Table S2, Fig. 2C). Although the roughness was slightly higher than that of the electrospun surface (Fig. 2B), this change did not affect platelet adhesion.

Uniform dispersion of nMH on the stent surface prevented excessive surface height elevation. Although the particle size of nMH was ≈300 nm, the measured roughness (94 nm) was lower due to partial embedding within the polymer matrix. This controlled roughness is beneficial, as surfaces with roughness >2 μm can promote platelet adhesion and hinder endothelial cell attachment [33]. The nanoparticle-based coating therefore minimized these risks while providing functional advantages: MH continuously neutralized acidic degradation products, and EVL effectively inhibited early SMC proliferation. ATR-FTIR analysis of BVS, MH, EVL, and BVS-AEM composites (Fig. S4) showed an O–H stretching vibration at 3419 cm^−1^ for MH, typical of hydroxyl groups [34]. In the composite, this peak slightly shifted to 3442 cm^−1^, indicating physical inclusion rather than chemical bonding. The characteristic C=O stretching band of EVL at 1740 cm^−1^ was masked by polymer overlap; thus, EVL incorporation was verified by HPLC. The loading amounts of EVL and MH were 144.6 μg and 71.81 μg, respectively, with an EVL loading efficiency of 12.6 % (Table S1), consistent with previous reports [33].

Drug-release studies demonstrated a biphasic EVL release profile, with ≈60 % released within 7 days followed by sustained release (Fig. 2D). This dual-phase pattern supports continuous suppression of VSMC proliferation while allowing re-endothelialization, as EVL was coated exclusively on the abluminal surface. In contrast, MH exhibited a linear release over 7 days (R^2^ = 0.9517, zero-order kinetics), whereas EVL followed first-order kinetics (R^2^ = 0.9805) (Table S2). The distinct release profiles reflect their different interactions with the PDLLA matrix [33]; the linear release of MH provides consistent buffering of acidic PLLA degradation products over time.

If the entire stent surface were uniformly coated, both endothelial and smooth-muscle cells would be inhibited, leading to poor endothelialization and potential thrombosis. In contrast, our abluminal-only coating preserved the luminal ALA layer's endothelial benefits while selectively suppressing SMC proliferation. This spatially controlled multilayer design effectively balances vascular healing and restenosis prevention, offering a significant advantage over conventional uniform-coating methods.

### 3.3 Characterization of outermost dot-printed DOX-loaded dMSN coating on biodegradable vascular stent

**Fig. 3:**
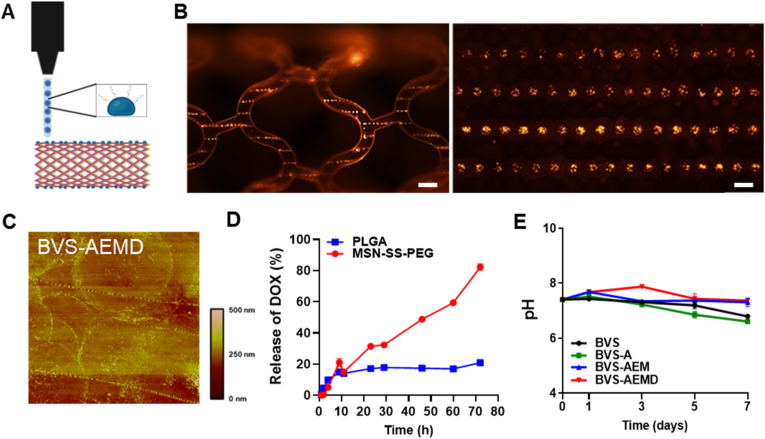
**Characterization of a coated BVS for controlled drug release at specific locations and times (BVS-AEMD):** (A) Schematic illustration of top-layer drug dot-printing using the ring-electrode nozzle system, (B) Fluorescence microscopy images showing uniform printing pattern (Ring-E system) (Scale bar = 100 μm), (C) AFM image of the coated BVS-AEMD surface (Scale bar = 10 μm), (D) Cumulative release profile of DOX from MSN-SS-PEG and PLGA nanoparticles at 37 °C for 75 h, and (E) pH variation of different BVS systems over 37 °C for 7 days.

Surface micropatterning technologies have recently gained attention for enhancing drug delivery precision and biocompatibility in bioresorbable stents [10]. Among these, precision dot printing offers efficient, low-waste, and spatially controllable drug deposition, making it a promising platform for tailored therapeutic delivery [[35], [36], [37]]. In this study, a ring-electrode nozzle system was used to print drug-loaded PEG-DA ink onto the outermost surface of PLLA-based stents (Fig. 3A). The resulting dot arrays were evaluated for uniformity, dimensional accuracy, surface roughness, and dual-drug coating feasibility.

TEM imaging (Fig. S7A) showed that dMSN–OH nanoparticles exhibited uniform spherical morphology with ordered mesopores and an average diameter of ∼200 nm, optimal for vascular delivery by enabling efficient endothelial uptake and high drug loading while minimizing platelet adhesion [38,39]. The particle size distribution (Fig. S7B) displayed a narrow peak around 100 nm, confirming uniformity. Nitrogen adsorption–desorption analysis (Fig. S8) revealed a type IV isotherm with a large specific surface area (∼580 m^2^ g^−1^) and uniform pore diameter (∼3.8 nm), indicating abundant adsorption sites and stable diffusion pathways for reproducible drug loading.

Stability assessments of MSN–OH and MSN@RhB–SS–PEG (Fig. S11) confirmed near-neutral pH (6.8–7.4) in PBS solution (37 °C) for 7 days, demonstrating chemical stability and minimal acidification. Particle size and zeta potential showed negligible change before and after drug loading (Fig. S7C), indicating successful incorporation without aggregation.

AFM analysis (Fig. 3C) confirmed precise and uniform deposition of the drug-loaded ink, producing evenly spaced, dimensionally consistent dots, while fluorescence imaging (Fig. 3B) verified homogeneous surface coverage. The ring-electrode configuration generated a symmetric electric field that minimized ink drift and droplet spreading, yielding sharper and more reproducible patterns than conventional setups.

The printed coating showed an average roughness (Rz) of 49.609 nm, comparable to pristine PLLA (Table S2), indicating that the process preserved surface smoothness. Such low roughness minimizes platelet and protein adhesion, reducing thrombosis and restenosis risks [33,34]. Collectively, these findings demonstrate that the ring-electrode dot printing system enables high-precision, biocompatible surface engineering suitable for bioresorbable stents.

For dual-drug printing, fluorescent model drugs rhodamine B (RB) and fluorescein dextran (FD) were separately incorporated into PEG-DA inks to assess droplet control. Under identical conditions, FD dots averaged ∼12 μm and RB dots ∼30 μm in diameter (Fig. 3B), confirming tunable droplet size via ink composition. The total DOX loading in BVS-AEMD was 23.56 μg (Fig. S7) [40].

Comparative release studies between MSN-SS-PEG and PLGA-PEG delivery systems over 70 h revealed distinct behaviors. To achieve sequential therapeutic regulation, DOX and EVL were designed with distinct temporal functions. The outermost PLGA–PEG layer was optimized for rapid DOX release within 3 days to suppress early inflammatory and hyperproliferative responses immediately after implantation, while EVL in the abluminal PDLLA/MH layer provided sustained release for more than 4 weeks to ensure long-term inhibition of smooth muscle proliferation. Considering the potent cytotoxicity of DOX, its short-term burst release was intentionally adopted to prevent endothelial damage and systemic toxicity while effectively suppressing early restenosis.

MSN–SS–PEG exhibited a rapid initial release, reaching approximately 80 % cumulative release within 70 h, indicating its suitability for early therapeutic intervention and efficient suppression of initial restenosis. In contrast, PLGA–PEG demonstrated a slower and more prolonged release profile (∼30 % cumulative at 70 h), supporting its use for long-term therapeutic maintenance. These results are consistent with the faster degradation rate of MSN–SS–PEG compared with PLGA–PEG (Fig. 3D). At 9 h, 14.12 % of DOX was released from PLGA and 19.94 % from MSN–SS–PEG; by 72 h, cumulative release reached 19.83 % (PLGA) and 77.98 % (MSN–SS–PEG), confirming MSN–SS–PEG's superior early-release capability. This rapid and efficient release behavior supports its role in acute-phase restenosis suppression, while PLGA–PEG and PDLLA/MH layers sustain long-term antiproliferative control [35].

To evaluate pH stability, coated PLLA samples were immersed in PBS solution at 37 °C for 7 days. As shown in Fig. 3E, all groups maintained a near-neutral pH (6.75–7.4) throughout the observation period, confirming that the multilayer coatings effectively prevented acid–base shifts and maintained environmental stability suitable for biological applications.

To further assess whether the coating layer affected the degradation behavior of the PLLA substrate, we monitored pH variation under accelerated conditions (PBS solution, 37 °C, 7 days). As shown in Fig. 3E, all groups maintained a stable, near-neutral pH (6.75–7.4) throughout the observation period. In groups without magnesium hydroxide (MH), a slight decrease in pH was observed, which can be attributed to the partial hydrolysis of the polymer components used in the coating solution. The absence of a marked pH decrease indicates that the release of acidic lactic-acid degradation products did not occur, suggesting that the PLLA substrate did not undergo accelerated hydrolysis. These results confirm that the coating layer did not affect the intrinsic degradation behavior of PLLA. This observation can be explained by the surface-erosion degradation mechanism of PLLA, in which hydrolysis proceeds gradually from the outer surface toward the interior [41]. Therefore, the MH-based coating layer functions primarily as a surface buffering and interfacial stabilization layer, rather than altering bulk polymer degradation.

Additionally, time-dependent pH variation in dMSN-containing solutions (Fig. S11) indicated that hydroxyl (–OH) groups on dMSNs interacted with free hydrogen ions to buffer localized acidity. This inherent buffering capability mitigates acidic microenvironments, enhances cell viability, and supports tissue regeneration. Since acidic conditions can impede vascular healing, the intrinsic pH-modulating ability of dMSNs contributes to improved biocompatibility and regenerative potential [36].

In summary, the ring-electrode dot-printing technique successfully achieved precise, uniform, and biocompatible drug deposition on bioresorbable stents. The integration of MSN–SS–PEG and PLGA–PEG delivery systems enabled spatiotemporal control of dual-drug release—rapid DOX delivery for early restenosis suppression and sustained EVL release for long-term vascular stabilization. The coatings maintained surface integrity, near-neutral pH, and intrinsic polymer stability, collectively demonstrating the potential of this hybrid surface-engineering strategy for next-generation multifunctional biodegradable vascular scaffolds [42].

### 3.4 Blood compatibility of the BVS-AEMD

**Fig. 4:**
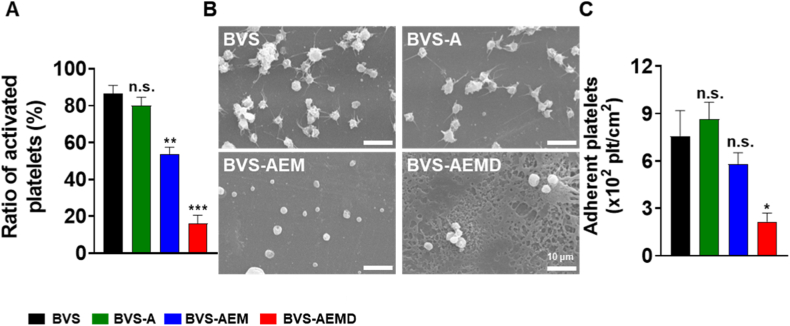
**Blood compatibility of the coated PLLA films:** (A) Quantification of adhered platelets on the surfaces of PLLA, ALA-modified PLLA (PLLA-A), AEM-coated PLLA (PLLA-AEM), and dual-coated PLLA (PLLA-AEMD), (B) SEM images of platelets adhered to the surfaces of PLLA, PLLA-A, PLLA-AEM, and PLLA-AEMD (Scale bar = 10 μm), and (C) Ratio of activated platelets on each surface (Values are presented as mean ± SD (n = 3/group) and statistical significance was determined using unpaired t-tests or one-way analysis of variance (ANOVA) with Tukey's multiple comparison post-test (∗*p* < 0.05; ∗∗*p* < 0.01; ∗∗∗*p* < 0.001; ∗∗∗∗*p* < 0.0001)).

Platelets play a critical role in thrombus formation by adhering to vascular injury sites, becoming activated, and aggregating. Although this physiological response is essential for hemostasis, it can cause severe complications such as thrombosis after stent implantation [43]. Therefore, suppressing platelet adhesion and activation remains a key challenge in the design of vascular stents. To evaluate the hemocompatibility of the coated surfaces, platelet adhesion and activation were quantitatively and qualitatively analyzed on PLLA films with different coatings. LDH assay results revealed platelet adhesion values of 7.58, 8.63, 2.15, and 5.81 for PLLA, PLLA-A, PLLA-AEM, and PLLA-AEMD, respectively. Among these, the PLLA-AEM group exhibited the lowest platelet attachment, indicating the strongest inhibitory effect (Fig. 4A).

SEM imaging provided further morphological evidence (Fig. 4B). Numerous filopodia—characteristic of activated platelets—were observed on the PLLA and PLLA-A surfaces, indicating high activation states. In contrast, round and non-activated platelets were predominantly observed on the PLLA-AEM surface, while the PLLA-AEMD surface also showed relatively low activation. The slight increase in surface roughness was attributed not to coating irregularities but to minor surface damage during the platelet fixation process.

Quantitative assessment of platelet activation showed ratios of 86.53 %, 80.12 %, 53.84 %, and 16.02 % for PLLA, PLLA-A, PLLA-AEM, and PLLA-AEMD, respectively. The PLLA-AEMD coating reduced platelet activation by approximately 5.4-fold compared with uncoated PLLA, demonstrating the most favorable anti-thrombotic performance (Fig. 4C).

This inhibitory behavior results from the combined effects of the coating components. Magnesium hydroxide (MH) enhances surface hydrophilicity, thereby reducing protein adsorption and platelet adhesion, while doxorubicin (DOX) interferes with platelet activation processes and mitigates pro-thrombotic signaling. Consequently, the PLLA-AEM and PLLA-AEMD coatings significantly improved hemocompatibility and reduced thrombogenicity compared with uncoated or ALA-only surfaces.

Although the PLLA-AEMD layer is located on the abluminal surface, it indirectly contributes to thromboresistance by maintaining surface smoothness and stabilizing the overall scaffold environment. The PLLA-A layer on the luminal surface also influenced platelet behavior, but the observed activation does not suggest a detrimental effect, as α-lipoic acid (ALA) itself possesses antithrombotic and antioxidant properties.

In summary, the incorporation of MH and DOX in the coating formulation not only improves surface physicochemical characteristics but also exerts biological effects that collectively suppress platelet adhesion and activation. This multifunctional coating strategy effectively minimizes thrombus formation and holds strong potential for clinical translation in next-generation biodegradable vascular scaffolds (BVS).

### 3.5 Protective effects of BVS-A on endothelial cells

**Fig. 5:**
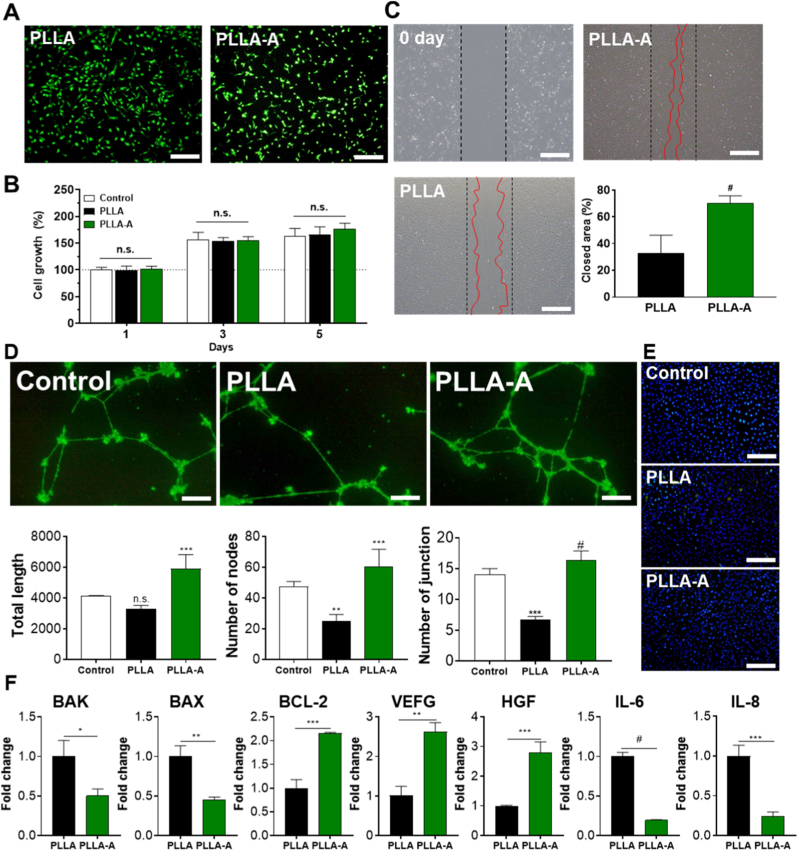
***In vitro* evaluation of improvement effect of endothelial cells:** (A) Live/dead staining images of HCAECs cultured on the scaffolds (Scale bar = 200 μm), (B) Cell proliferation of HCAECs on the scaffold surfaces, (C) Cell migration quantified by the wound healing assay (closed area) (Scale bar = 200 μm), (D) Tube formation assay: fluorescence images of calcein AM-stained endothelial structures (scale bar: 500 μm), (E) Representative immunofluorescence images showing IL-8 expression in TNF-α-pretreated HCAECs incubated with scaffolds (Scale bar = 100 μm), and (F) mRNA expression levels of cell growth-related genes (Values are presented as mean ± SD (n = 3), and statistical significance was determined using unpaired t-tests or one-way analysis of variance (ANOVA) with Tukey's multiple comparison post-test ∗*p* < 0.05; ∗∗*p* < 0.01; ∗∗∗*p* < 0.001; ∗∗∗∗*p* < 0.0001; ns: no significant difference).

Vascular stents promote vascular healing by interacting with endothelial cells, helping to prevent complications such as restenosis and thrombosis. Fig. 5 presents the biological responses of human coronary artery endothelial cells (HCAECs) to the ALA-coated BVS (BVS-A). Cell viability and cytotoxicity were first evaluated using Live/Dead staining and conditioned medium assays. As shown in Fig. 5A, there were no significant differences in cell proliferation between PLLA and BVS-A surfaces, and no cytotoxic effects were observed. Conditioned media collected after three days of scaffold incubation also exhibited no toxicity toward HCAECs (Fig. 5B), confirming the biocompatibility of ALA release. Cell migration was assessed using a wound-healing assay (Fig. 5C). Over time, the wound area decreased in all groups (Control, PLLA, and BVS-A), but the BVS-A group showed a 2.16-fold greater migration distance compared with PLLA. These results indicate that ALA enhances endothelial motility, likely through its antioxidant activity and modulation of migration-related signaling pathways, thereby promoting re-endothelialization, a critical process for rapid endothelial coverage after implantation [38].

To investigate inflammatory responses, immunocytochemistry staining for IL-8, an early-stage inflammatory cytokine, was performed following TNF-α stimulation. IL-8 plays a key role in recruiting neutrophils during the early phase of inflammation. While a slight increase in IL-8 expression was observed in the PLLA group, BVS-A markedly reduced fluorescence intensity, indicating a strong anti-inflammatory effect (Fig. 5E). These findings are consistent with previous studies showing that ALA suppresses pro-inflammatory cytokine production and reduces oxidative stress [44].

The angiogenic potential of the endothelial cells was further examined using a tube formation assay (Fig. 5D). The control group exhibited moderate tube-like network formation, whereas the PLLA group showed reduced network complexity, suggesting impaired angiogenic activity caused by acidic degradation products of PLLA. In contrast, BVS-A significantly restored tubular structure formation, with enhanced branching and interconnectivity. Quantitative analysis of total mesh area, number of junctions, and total segment length supported these observations, showing that ALA coating promotes endothelial network formation and vascular regeneration. These results imply that ALA exerts pro-angiogenic effects by modulating endothelial behavior under oxidative stress [45]. [45].

ALA is known to activate PI3K/Akt signaling and enhance endothelial nitric oxide synthase (eNOS) activity, both of which are critical for angiogenesis. The observed increase in branching and network complexity in the PLLA-A group suggests improved endothelial migration, alignment, and intercellular communication—key steps in neovessel formation. [[46], [47], [48]]. In agreement with previous findings on the antioxidant and vasoprotective functions of ALA, the present results demonstrate that ALA not only mitigates oxidative injury but also supports capillary-like structure formation.

To further confirm these biological effects, quantitative RT-PCR was conducted to analyze inflammation-, apoptosis-, and angiogenesis-related gene expression (Fig. 5F). The expression of pro-apoptotic markers (BAK and BAX) was significantly downregulated in the ALA-coated group compared with uncoated PLLA, while anti-apoptotic BCL-2 expression was increased, suggesting that ALA provides cytoprotective effects through BCL-2–mediated pathways. In the PLLA group, IL-8 fluorescence was strongly elevated, reflecting oxidative stress and inflammation caused by PLLA degradation. In contrast, the BVS-A group showed a marked reduction in IL-8 signal intensity, consistent with the suppression of pro-inflammatory cytokines.

These findings correlate with the DPPH radical scavenging results shown in Fig. 5E, demonstrating that ALA effectively eliminates ROS at the cellular level, thereby reducing the expression of inflammatory cytokines such as IL-6 and IL-8. Concurrently, angiogenesis-related genes VEGF and HGF were significantly upregulated, implying enhanced neovascularization and tissue regeneration. The increased expression of HGF, a key regulator of repair processes, further supports the role of ALA in promoting wound healing and tissue remodeling.

Collectively, these results indicate that ALA coating enhances the endothelial microenvironment not solely through anti-apoptotic signaling but through the combined suppression of oxidative stress and inflammation, while simultaneously promoting angiogenic and regenerative functions [[49], [50], [51]]. [[49], [50], [51]]. The upregulation of VEGF and HGF supports the idea that ALA improves angiogenic signaling and hypoxia resilience, contributing to better tissue integration and perfusion.

In summary, BVS-A exhibited multifaceted protective effects on endothelial cells, including enhanced survival, migration, and angiogenic activity, along with reduced inflammatory and apoptotic responses. These findings highlight ALA-functionalized BVS as a promising approach for next-generation vascular stents with superior endothelial compatibility and long-term vascular healing outcomes.

### 3.6 ROS scavenging effect of BVS-A

**Fig. 6:**
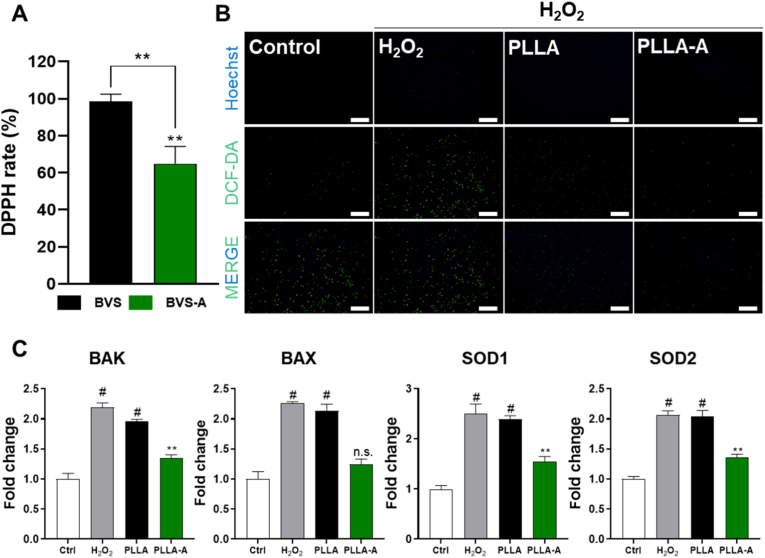
**Antioxidant and ROS-regulatory effects of ALA-coated PLLA:** (A) DPPH radical scavenging activity of the samples, (B) Intracellular ROS levels in HCAECs visualized by DCF-DA fluorescence staining (Scale bar = 100 μm), and (C) mRNA expression levels of ROS-related genes in HCAECs (Values are presented as mean ± SD (n = 3/group), and statistical significance was determined using unpaired t-tests or one-way analysis of variance (ANOVA) with Tukey's multiple comparison post-test ∗∗*p* < 0.01; ∗∗∗∗*p* < 0.0001; ns: no significant difference).

Oxidative stress arises from excessive accumulation of reactive oxygen species (ROS) within cells, which is a major contributing factor to cellular dysfunction, inflammatory responses, and apoptosis. In particular, vascular endothelial cells are highly sensitive to external stimuli, and prolonged exposure to oxidative stress can lead to endothelial dysfunction and progression of vascular lesions. Therefore, if a biomaterial can effectively eliminate ROS and induce antioxidant defense mechanisms, it may promote endothelial cell survival and functional recovery in damaged vascular environments [[52], [53], [54], [55]]. [[52], [53], [54], [55]]. In Fig. 6, we developed a BVS-A by coating PLLA with ALA, a well-known antioxidant [56], and evaluated its protective effects against oxidative stress *in vitro*. The antioxidant capacity of BVS-A was comprehensively analyzed through various indicators, including DPPH radical scavenging activity, intracellular ROS fluorescence measurement, and antioxidant gene expression analysis. DPPH analysis revealed that the radical scavenging activity of the PLLA-A increased by 33.68 % compared to uncoated PLLA (Fig. 6A), indicating that the antioxidant property of ALA was successfully integrated into the film. This suggests that ALA effectively enhances the antioxidative profile of PLLA and contributes to the reduction of ROS accumulation. Under oxidative stress conditions induced by H_2_O_2_ treatment, intracellular ROS levels were assessed using the DCF-DA fluorescent probe. The BVS group exhibited a slight reduction in ROS-derived fluorescence intensity, likely due to the intrinsic stability of PLLA and surface treatment effects. Notably, the BVS-A group showed a significantly greater decrease in ROS-derived fluorescence compared to the BVS group (Fig. 6B), demonstrating that ALA coating effectively suppresses ROS generation or scavenges existing ROS, thereby alleviating oxidative damage at the cellular level. Consistently, RT-qPCR analysis of antioxidant-related gene expression revealed significant upregulation of SOD1 and SOD2 in the BVS-A group (Fig. 6C). These enzymes play critical roles in neutralizing ROS. The observed upregulation aligns with previous reports suggesting that ALA activates the endogenous antioxidant response, potentially via the NRF2-ARE signaling pathway [57,58]. Furthermore, differences were observed in the expression of apoptosis-related genes. The BVS-A group exhibited downregulation of apoptotic markers BAX and BAK, indicating that ALA effectively suppresses apoptosis associated with oxidative stress. In addition to its intrinsic antioxidant activity, ALA can be converted into its reduced form, dihydrolipoic acid (DHLA), which plays a central role in the cellular antioxidant network by regenerating endogenous antioxidants such as glutathione, vitamin C, and vitamin E [59]. The observed reduction in ROS, upregulation of antioxidant genes, and inhibition of inflammation and apoptosis in this study reflect these multifunctional biological properties of ALA [60,61]. Collectively, BVS-A is evaluated as a multifunctional biomaterial capable of protecting vascular endothelial cells from oxidative stress by eliminating ROS, enhancing endogenous antioxidant defenses, and suppressing inflammatory and apoptotic pathways [62]. These findings suggest that BVS-A holds promise as a therapeutic strategy in various pathological conditions accompanied by oxidative stress, including vascular injury and ischemic environments.

### 3.7 Inhibition effects on smooth muscle cells of the BVS-AEMD

**Fig. 7:**
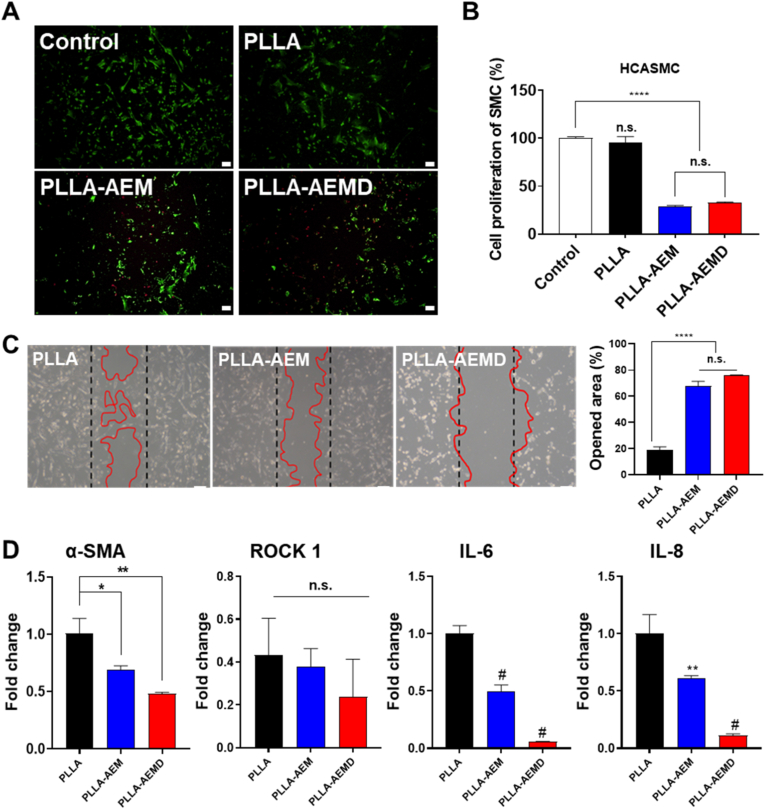
**Inhibition effects on smooth muscle cells of the BVS-AEMD:** (A) Live/dead staining images (Scale bar = 200 μm), (B) Cell proliferation of HCASMCs to scaffold, (C) Cell migration detected by wound healing assay closed area (Scale bar = 200 μm), and (D) The mRNA expression levels of cell growth genes (Values are presented as mean ± SD (n = 3), and statistical significance was determined using unpaired t-tests or one-way analysis of variance (ANOVA) with Tukey's multiple comparison post-test ∗*p* < 0.05; ∗∗*p* < 0.01; ∗∗∗∗*p* < 0.0001; ns: no significant difference).

HCASMCs are a major contributor to in-stent restenosis (ISR) following stent implantation. Proliferation and migration of HCASMCs on BVS play a critical role in neointimal formation [63]. [63]. To address this, we developed BVS-AEMD, in which everolimus (EVL), an inhibitor of cell cycle progression and DNA synthesis, was applied to the second layer of the BVS, while DOX, a cytotoxic agent with vasoconstrictive properties, was applied to the outermost layer to suppress early-stage restenosis [64]. The effects of this dual-drug coating on HCASMCs proliferation were evaluated using live/dead staining and CCK-8 assays (Fig. 7A and B). Compared to the control and PLLA-only groups, the EVL and DOX-treated group exhibited a marked reduction in calcein AM-positive viable cells, indicating significantly reduced cell proliferation. The CCK-8 assay further confirmed this result, showing approximately 66.87 % inhibition of cell proliferation in the dual-drug group. To assess the effect of drug coatings on cell migration, cell motility was evaluated through a wound healing assay (Fig. 7C). The PLLA group exhibited active migration, nearly closing the wound area, whereas the EVL- and Dox-coated groups (PLLA-AEM and PLLA-AEMD) showed significantly suppressed migration. In particular, the PLLA-AEMD group demonstrated only 24.14 % wound closure, indicating potent inhibition of cell motility by the dual-drug coating. To investigate the underlying mechanisms, we analyzed the expression of key markers associated with smooth muscle cell function, including α-SMA and ROCK1 (Fig. 7D). DOX significantly downregulated α-SMA expression by inhibiting the TGF-β signaling pathway and also reduced the expression of ROCK1, a key component of the RhoA-ROCK pathway, thereby suppressing cell contraction and migration. EVL, while less potent in reducing α-SMA levels, effectively inhibited cell proliferation via the mTOR pathway and also reduced ROCK1 expression, contributing to the dual effect of restenosis prevention and vascular healing. These findings suggest that the dual-drug strategy combining mTOR inhibition by EVL and suppression of α-SMA and ROCK1 by DOX effectively regulates both proliferation and migration of HCASMCs via complementary mechanisms. This synergistic regulation enhances the anti-inflammatory and *anti*-restenotic properties of the BVS. Moreover, previous studies have shown that ROCK1 inhibition reduces caspase activation and necrotic cell death, suggesting that Dox may not only exert cytotoxic effects but also modulate cell survival responses under pathological conditions. Therefore, the combination of Dox and EVL offers a promising approach to simultaneously suppress pathological HCASMCs behaviors while maintaining sufficient cell proliferation, thereby improving the overall biocompatibility and function of BVS.

In addition, the temporal drug-release design of the BVS-AEMD scaffold further enhances this therapeutic synergy. As shown in Fig. 7C, DOX exhibits strong cytotoxic activity, effectively removing hyperplastic and inflammatory cells during the early stage after implantation. To avoid prolonged toxicity to endothelial cells, DOX was dot-printed on the outermost surface for rapid burst release, thereby targeting only early restenosis. Subsequently, the MH layer neutralizes lactic acid produced by PLLA degradation, maintaining pH stability and preventing inflammatory activation, while EVL provides a sustained antiproliferative effect to prevent late-stage restenosis. This sequential therapeutic coordination early DOX release, mid-phase MH buffering, and long-term EVL suppression achieves a temporally balanced healing response, combining early inhibition of inflammation with long-term vascular regeneration.

### *3.8* *In vivo* evaluation of the BVS-AEMD after implantation

**Fig. 8:**
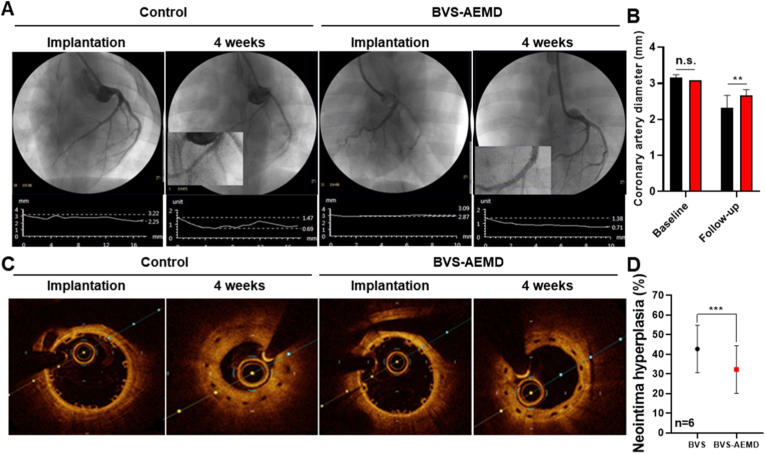
***In vivo* analysis of soluble polymer fusion scaffolds for cardiovascular intervention:** (A) Representative angiographic images of coronary arteries after implantation and at 4 weeks in control and BVS-AEMD groups, (B) Quantitative analysis of coronary artery diameters at baseline and 4 weeks post-implantation, (C) Representative optical coherence tomography (OCT) cross-sectional images at baseline and 4 weeks showing neointimal formation (Scale bar = 200 μm), and (D) Quantification of neointimal hyperplasia (%) in BVS and BVS-AEMD groups (Values are presented as mean ± SD (n = 15), and statistical significance was determined using unpaired t-tests or one-way analysis of variance (ANOVA) with Tukey's multiple comparison post-test ∗∗*p* < 0.01; ∗∗∗*p* < 0.001; ∗∗∗∗*p* < 0.0001; ns: no significant difference).

To evaluate the *in vivo anti*-restenotic efficacy of the BVS-AEMD scaffold, coronary angiography and optical coherence tomography (OCT) were performed at baseline and 4 weeks post-implantation in a porcine coronary artery model. Coronary angiographic images (Fig. 8A) revealed comparable arterial diameters between the control and BVS-AEMD groups immediately after implantation. However, at the 4 weeks follow-up, the control group exhibited a significant reduction in vessel diameter, indicating progression of restenosis, while the BVS-AEMD group maintained a stable vessel diameter. Quantitative angiographic analysis (Fig. 8B) supported these findings; although there were no significant differences at baseline, the BVS-AEMD group showed a significantly larger luminal area than the control group at 4 weeks, confirming the scaffold's superior lumen-preserving capability. Similar trends were observed in OCT image analysis (Fig. 8C). Both groups exhibited comparable vascular morphology at baseline. However, after 4 weeks, the control group showed marked neointimal thickening and luminal narrowing, indicative of substantial neointimal hyperplasia. In contrast, the BVS-AEMD group demonstrated a thinner neointimal layer and a more preserved lumen. Quantitative OCT analysis (Fig. 8D) showed that the luminal diameter of the BVS-AEMD group (2.21 ± 0.10 mm) was significantly larger than that of the control group (1.95 ± 0.14 mm, *p* < 0.01), and the neointimal hyperplasia percentage was markedly reduced (24.8 ± 3.5 % vs. 41.2 ± 6.1 %, *p* < 0.001). (Fig. 8D). Notably, the 4 weeks' time point holds clinical relevance, as large-scale patient cohort studies have reported that the majority of thrombotic occlusion and major adverse cardiac events after coronary stent implantation occur within the first 4 weeks [65]. These *in vivo* findings are consistent with the *in vitro* results, where the BVS-AEMD scaffold effectively inhibited HCASMC proliferation and migration. The dual-drug design of the scaffold appears to function effectively in a physiological environment. Everolimus (EVL), coated on the intermediate layer of the scaffold, inhibits cell proliferation and survival signaling through the mTOR pathway [66]. Meanwhile, doxorubicin (Dox), applied to the outermost layer, regulates cell migration, contractility, and inflammation via inhibition of the TGF-β and RhoA-ROCK pathways [67]. Notably, suppression of ROCK1 expression is also associated with reduced cell death and inflammatory responses, suggesting that BVS-AEMD may contribute not only to anti-proliferative effects but also to a more favorable vascular healing microenvironment. Conventional drug-eluting stents, primarily relying on a single drug such as EVL or sirolimus, have been reported to suffer from delayed re-endothelialization and an increased risk of long-term restenosis compared to newer strategies. In contrast, the BVS-AEMD system employs a multi-drug, multilayer coating approach, enabling temporally and spatially controlled drug release. This design not only accelerates endothelial recovery but also effectively suppresses smooth muscle cell proliferation, demonstrating superior vascular healing compared to conventional DES or alternative drug delivery methods [68]. The BVS-AEMD scaffold proposed in this study incorporates a dual-drug strategy using a biodegradable matrix, which enables simultaneous control of early inflammatory responses and long-term inhibition of smooth muscle cell activity. This approach offers a promising alternative to overcome limitations of current stent systems.

Although a direct side-by-side comparison with metallic DESs was not included in this study, such comparison would be confounded by fundamental material and structural differences. Metallic DESs, typically possessing thinner struts (∼80 μm) and permanent frameworks, differ substantially from biodegradable PLLA-based scaffolds (∼150 μm) that gradually resorb and restore vascular physiology. These intrinsic differences significantly affect implantation success, degradation behavior, and vascular healing kinetics. Therefore, the present work focused on comparative evaluation within the BVS platform to highlight the functional benefits of the multilayer AEMD design. Notably, the BVS-AEMD system demonstrates key advantages inherent to biodegradable scaffolds complete resorption, minimized chronic inflammation, and temporally controlled drug release which collectively represent a complementary rather than directly competing strategy to existing DES technologies.

### *3.9* *In vivo* evaluation of functionalized BVS-AEMD

**Fig. 9:**
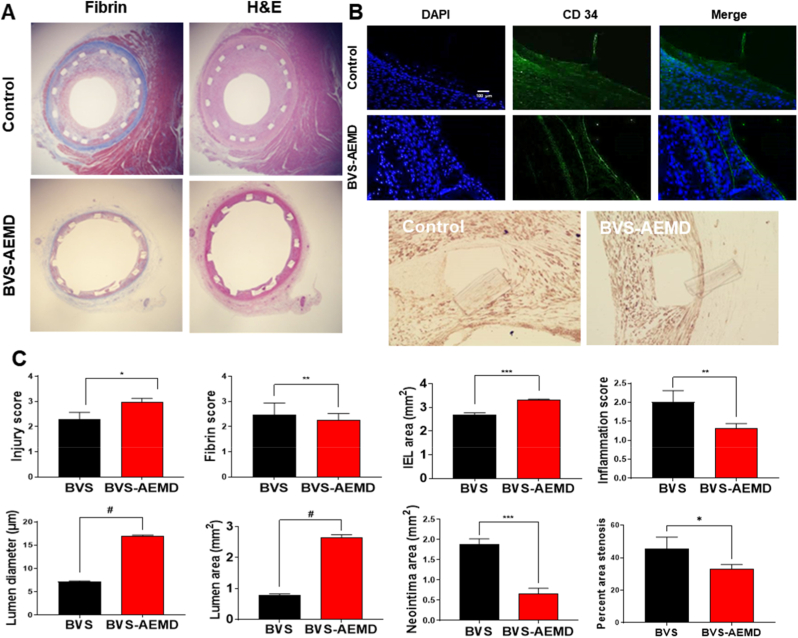
***In vivo* evaluation of BVS-AEMD after implantation:** (A) Tissue staining images around the struts of the soluble polymer fusion scaffold for cardiovascular intervention: Fibrin stain and H&E (Scale bar = 200 μm), (B) Immunofluorescence (IF) and immunohistochemistry (IHC) staining images. CD34 expression was confirmed through IF staining, and alpha smooth muscle cell expression was verified using IHC DAB (Scale bar = 100 μm), and (C) Quantitative biological evaluation based on OCT and histological analysis: injury score, fibrin score, inflammation score, IEL area, lumen diameter, lumen area, and neointima area (Values are presented as mean ± SD (n = 15), and statistical significance was determined using unpaired t-tests or one-way analysis of variance (ANOVA) with Tukey's multiple comparison post-test ∗*p* < 0.05; ∗∗*p* < 0.01; ∗∗∗*p* < 0.001; ∗∗∗∗*p* < 0.0001).

Histological and morphometric evaluations were conducted 4 weeks after implantation to assess the vascular responses induced by the BVS-AEMD scaffold compared with the control BVS (Fig. 9). Fig. 9A shows histological staining of vascular cross-sections. In these images, the vascular lumen in the BVS-AEMD scaffold appears visibly wider and more clearly preserved than that of the control BVS, indicating improved luminal patency and reduced neointimal encroachment. In addition, histological staining revealed fibrin deposition and inflammatory cell infiltration around the scaffold struts in both groups, indicating active healing and remodeling processes following implantation. However, the density and extent of fibrin and inflammatory infiltration appeared less pronounced in the BVS-AEMD scaffold, suggesting a relatively quiescent healing response.

As shown in Fig. 9B, immunofluorescence staining revealed distinct cellular responses between two groups, particularly in endothelial coverage and smooth muscle activation. CD34 staining revealed positive endothelial cell signals along the luminal surface in both groups, but the control group exhibited incomplete and discontinuous endothelial coverage. In contrast, the BVS-AEMD group displayed continuous and uniform CD34 expression, indicating more advanced endothelial restoration. Meanwhile, α-SMA staining, reflecting smooth muscle proliferation, appeared weaker and less extensive in the BVS-AEMD group, suggesting effective suppression of neointimal hyperplasia and more favorable vascular remodeling.

The underlying mechanism of this therapeutic advantage can be attributed to the synergistic interactions among ALA, MH, EVL, and DOX within the multilayer system. Beyond the individual pharmacological effects of ALA, MH, EVL, and DOX, the therapeutic outcome of the BVS-AEMD scaffold arises from their synergistic interplay. Early DOX release suppresses acute inflammation and removes hyperplastic cells, allowing subsequent ALA- and MH-mediated stabilization of the oxidative and acidic microenvironment. The buffering function of MH maintains EVL stability and supports its sustained antiproliferative efficacy, while ALA counteracts the transient inhibitory influence of EVL on endothelial proliferation through antioxidative and nitric-oxide–enhancing actions. These complementary and temporally coordinated effects establish a functional equilibrium between endothelial recovery and smooth-muscle suppression, resulting in selective and time-balanced vascular healing as demonstrated *in vivo.*

Fig. 9C presents the quantitative analysis based on the histological and immunofluorescence observations shown in Fig. 9A and B. The injury score, representing endothelial and medial disruption caused by implantation, was slightly higher in the BVS-AEMD scaffold (2.96 ± 0.152, *p* < 0.05) than in the control BVS (2.30 ± 0.245), likely due to localized procedural effects rather than pathological damage. The fibrin score was marginally lower in the BVS-AEMD scaffold (2.26 ± 0.25 vs. 2.46 ± 0.47, *p* < 0.01) reflecting faster fibrin clearance and improved surface healing. The inflammation score was markedly reduced (1.31 ± 0.125 vs. 2.01 ± 0.30, *p* < 0.01), indicating attenuation of immune-cell infiltration and resolution of chronic inflammation. Morphometric measurements further confirmed structural improvements: the internal elastic lamina (IEL) area increased (3.30 ± 0.041 mm^2^ vs. 2.68 ± 0.09 mm^2^, *p <* 0.001) the lumen diameter and area were significantly larger (16.90 ± 0.249 μm and 2.64 ± 0.09 mm^2^ vs. 7.13 ± 0.19 μm and 0.79 ± 0.03 mm^2^, *p* < 0.0001) and the neointima area decreased substantially (0.65 ± 0.13 mm^2^ vs. 1.89 ± 0.12 mm^2^, *p* < 0.001). Together, these quantitative results validate the histological and cellular observations, confirming that the BVS-AEMD scaffold effectively maintains luminal integrity while suppressing restenosis-associated remodeling.

Collectively, these results demonstrate that the BVS-AEMD scaffold effectively modulated early vascular responses by reducing vessel wall injury, fibrin accumulation, and inflammation, while promoting re-endothelialization and preserving luminal integrity. This multifaceted therapeutic performance stems from the spatiotemporally coordinated synergy among the incorporated components rather than their individual pharmacological roles. At the early phase of implantation, rapid DOX release suppresses acute inflammation and eliminates hyperplastic cells, creating a quiescent environment for tissue repair. Subsequently, ALA and MH act cooperatively to stabilize the oxidative and acidic microenvironment, mitigating ROS accumulation and preventing the local acidosis typically caused by PLLA degradation. The stabilized milieu further enhances EVL performance, enabling sustained antiproliferative efficacy without compromising endothelial recovery. Meanwhile, ALA counterbalances the transient inhibitory influence of EVL on endothelial proliferation through its antioxidative and nitric oxide–enhancing actions. Through this cascade of complementary and temporally aligned effects, the BVS-AEMD scaffold achieves a functional equilibrium between endothelial regeneration and smooth-muscle suppression, resulting in selective and time-balanced vascular healing as demonstrated *in vivo*. Unlike conventional DES that rely on a single antiproliferative agent [20,69], the multilayer BVS-AEMD system integrates early anti-inflammatory and antioxidative activity with sustained suppression of neointimal proliferation. The 4 weeks porcine model represents a critical phase of vascular remodeling, and the observed inhibition of inflammation and smooth-muscle proliferation provides strong prognostic evidence for long-term prevention of restenosis, although extended follow-up will be necessary to confirm chronic healing and degradation outcomes.

## 4 Conclusion

Multifunctional BVS-AEMD incorporating ALA, MH, EVL, and DOX has been successfully developed through a multilayer coating strategy. This design addresses the limitations of conventional stents by achieving both spatial selectivity and temporal control of therapeutic delivery tailored to distinct vascular cell types. The luminal ALA layer significantly promoted endothelial cell migration and angiogenic gene expression while reducing oxidative stress and inflammation, potentially involving the NRF2 pathway as suggested by previous studies. The abluminal layer containing MH and EVL effectively neutralized acidic degradation byproducts of PLLA and inhibited smooth muscle cell proliferation and migration, thereby mitigating the risk of in-stent restenosis. In addition, precision dot printing of DOX on the outermost layer provided localized suppression of inflammatory and contractile smooth muscle activity via inhibition of the TGF-β and RhoA/ROCK signaling pathways. *In vitro* studies confirmed enhanced hemocompatibility, reduced platelet activation, and selective regulation of endothelial and smooth muscle cells. *In vivo* evaluation using a porcine coronary artery model further demonstrated preserved luminal area, accelerated endothelialization, and effective suppression of neointimal hyperplasia and inflammatory responses. Collectively, the BVS-AEMD scaffold exhibits synergistic antioxidant, anti-inflammatory, anti-proliferative, and pro-endothelial properties, representing a promising therapeutic platform for next-generation vascular stents. Importantly, its multilayer design and spatiotemporally controlled drug release strategy provide a balanced approach that simultaneously promotes rapid endothelial recovery while preventing long-term restenosis. This paradigm highlights the potential of engineered bioresorbable scaffolds to improve long-term vascular healing and clinical outcomes beyond the capabilities of current drug-eluting stents.

## CRediT authorship contribution statement

**Duck Hyun Song:** Writing – original draft, Visualization, Validation, Resources, Project administration, Methodology, Investigation, Formal analysis, Data curation, Conceptualization. **Seungwoon Baik:** Writing – review & editing, Project administration, Data curation, Conceptualization. **Jun Yong Kim:** Writing – review & editing, Investigation, Formal analysis, Data curation. **Jeong min Park:** Investigation. **Byeongseok Ryu:** Investigation, Data curation. **Il Ho Seo:** Investigation. **Su Sam Lee:** Investigation. **Han Byul Kim:** Investigation. **Young Joon Hong:** Investigation. **Won-Gun Koh:** Supervision, Data curation. **WonHyoung Ryu:** Supervision, Data curation. **Yeu-Chun Kim:** Supervision. **Dong Ryul Lee:** Supervision. **Dong Keun Han:** Writing – review & editing, Supervision, Resources, Funding acquisition.

## Declaration of competing interest

The authors declare the following financial interests/personal relationships which may be considered as potential competing interests: Dong Keun Han reports financial support was provided by Korea Medical Device Development Grant funded by the Korean government. Dong Keun Han reports financial support was provided by Korean Fund for Regenerative Medicine (KFRM). Dong Keun Han reports financial support was provided by National Research Foundation of Korea. If there are other authors, they declare that they have no known competing financial interests or personal relationships that could have appeared to influence the work reported in this paper.

## Data Availability

Data will be made available on request.
