# Supplementary material for: Multilayer surface coating for enhanced anti-inflammation, anti-restenosis, and re-endothelialization in advanced biodegradable vascular stents
Source: Mater Today Bio. 2025 Nov 21;35:102570. doi: 10.1016/j.mtbio.2025.102570 (PMC12681855; doi:10.1016/j.mtbio.2025.102570)
Supplement: Multimedia component 1 [file mmc1.docx]

**Multilayer Surface Coating for Enhanced Anti-inflammation, Anti-restenosis, and Re-endothelialization in Advanced Biodegradable Vascular Stents**

**Duck Hyun Song^a,^**†**, Seungwoon Baik^b,^**†**,** [**Jun Yong Kim**](https://journals.sagepub.com/doi/full/10.1177/20417314211008626#con1)**^a,^**†**, Jeong min Park^a^, Byeongseok Ryu^c^, Il Ho Seo^d^, Su Sam Lee^e^, Han Byul Kim^f^, Young Joon Hong^g^, Won-Gun Koh^c^, WonHyoung Ryu^d^, Yeu-Chun Kim^e^, Dong Ryul Lee^a^, and Dong Keun Han^b*^**

^a^Department of Biomedical Science, CHA University, Korea

^b^ORANDBIO Co., Ltd., Korea

^c^Department of Chemical and Biomolecular Engineering, Yonsei University, Korea

^d^Department of Mechanical Engineering, Yonsei University, Korea

^e^Department of Chemical & Biomolecular Engineering, Korea Advanced Institute of Science and Technology, Korea

^f^Division of Radiation Biomedical Research, Korea Institute of Radiological and Medical Science, Seoul, Korea

^g^Division of Cardiology, Chonnam National University Hospital, Korea

2025. 11. 17

***Resubmitted to Materials Today Bio***

*****Correspondence: Dr. Dong Keun Han, dkhan618@gmail.com

†These authors contributed equally to this work.

Supplementary Material

**Table S1.** Drug composition, loading amount, and loading efficiency of each sample group

**
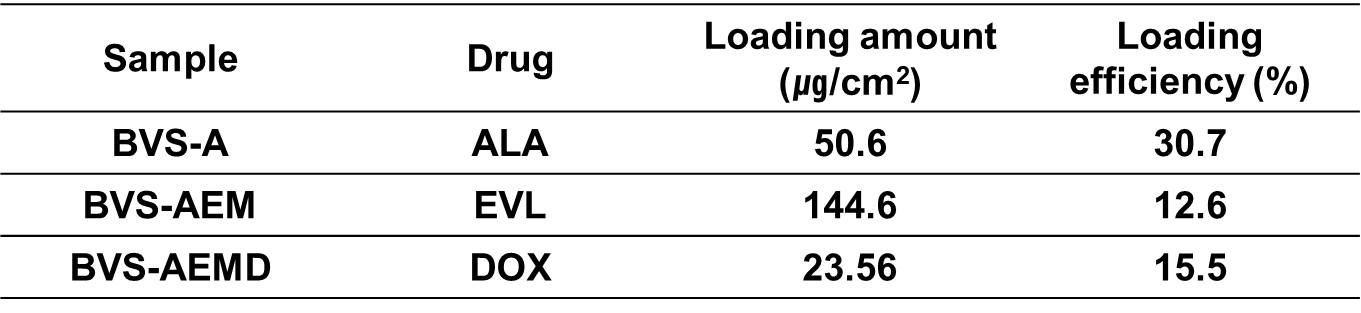
**

**Table S2.** AFM Measurements of PLLA-A, PLLA-AEM, and PLLA-AEMD

**
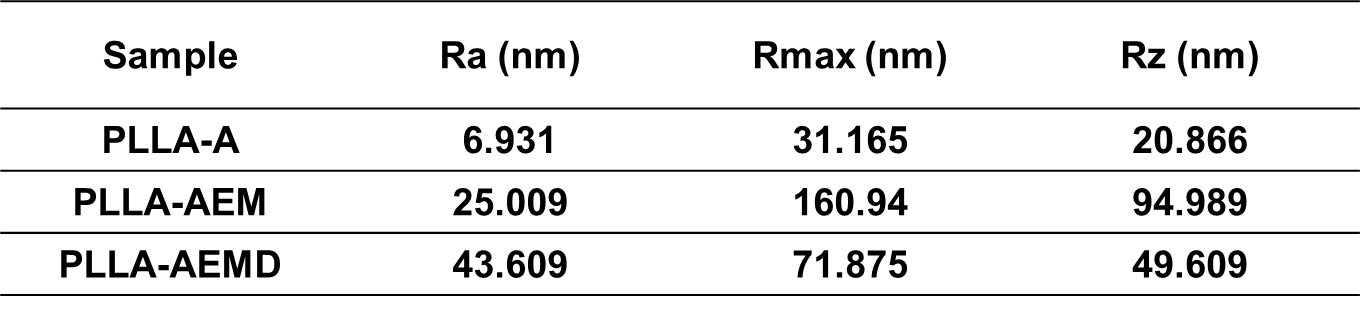
**

**Section S1.** Optimization of Fabrication Parameters

(a) Optimization of EHD Dot-Printing Parameters
Various voltages (8–12 kV), flow rates (0.5–2 mL h⁻¹), and nozzle-to-collector distances (10–20 cm) were evaluated to balance droplet stability and spatial uniformity. A voltage of 10 kV and 15 cm distance minimized jet instability and produced uniform 10 µm dot arrays without overspray. Pattern spacing (20–50 µm) was tuned to modulate diffusion-driven drug release, with 30 µm spacing yielding an optimal cumulative release (~70% over 72 h).

(b) Optimization of PLGA–PEG Synthesis

The PEG molecular weight (2 k–10 kDa) and feed ratios were varied to adjust hydrophilicity and degradation rate. A 1:1 (w/w) ratio of PLGA–NHS to mPEG–NH₂ yielded nanoparticles with stable dispersion (ζ ≈ –22 mV) and sustained DOX release. A 24 h reaction time ensured complete amide coupling while minimizing chain scission and viscosity loss.

(c) Synthesis of pyridyldithiol-terminated nanoparticles (dMSN-SS-PD):
dMSN-SH (250 mg) was dispersed in 25 mL methanol and slowly added into 10 mL of methanol containing 0.55 g 2,2′-dipyridyldisulfide and 0.2 mL glacial acetic acid. The mixture was stirred in the dark at room temperature for 24 h. The resulting dMSN-SS-PD nanoparticles were collected by centrifugation (15 000 rpm, 15 min) and washed with methanol three times.

(d) Synthesis of DOX-loaded MSN-SS-PEG (dMSN@DOX-SS-PEG):
dMSN-SS-PD (100 mg) was mixed with 25 mL of DOX solution (2 mg mL⁻¹ in PBS) and sonicated for 5 min to obtain a well-dispersed suspension. The mixture was stirred at room temperature in the dark for 24 h, followed by the addition of 100 mg thiolated PEG. After another 24 h of reaction, the suspension was centrifuged (15 000 rpm, 15 min), washed thoroughly with PBS (pH 7.4) until the supernatant became colorless, and vacuum-dried for further use.


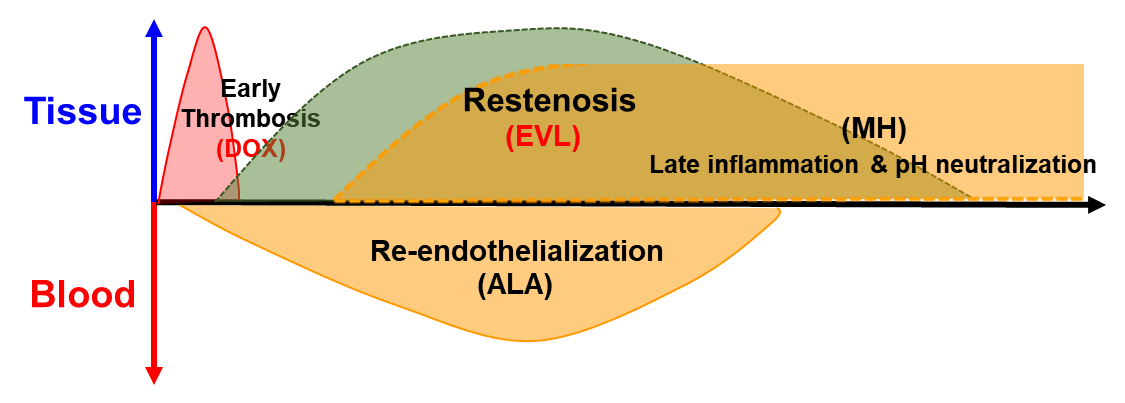


**Fig. S1.** Sequential release and therapeutic phases of the BVS-AEMD system.


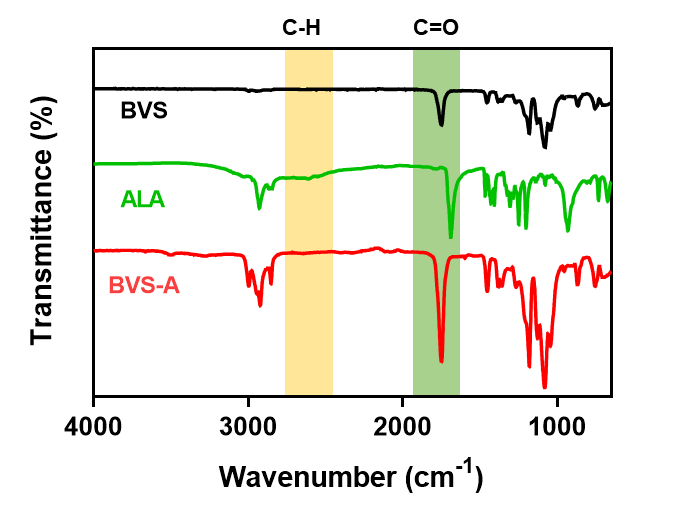


**Fig. S2.** ATR-FTIR spectra of unmodified BVS, pure ALA, and BVS-A formulations in BVS coated with ALA (BVS-A).

**
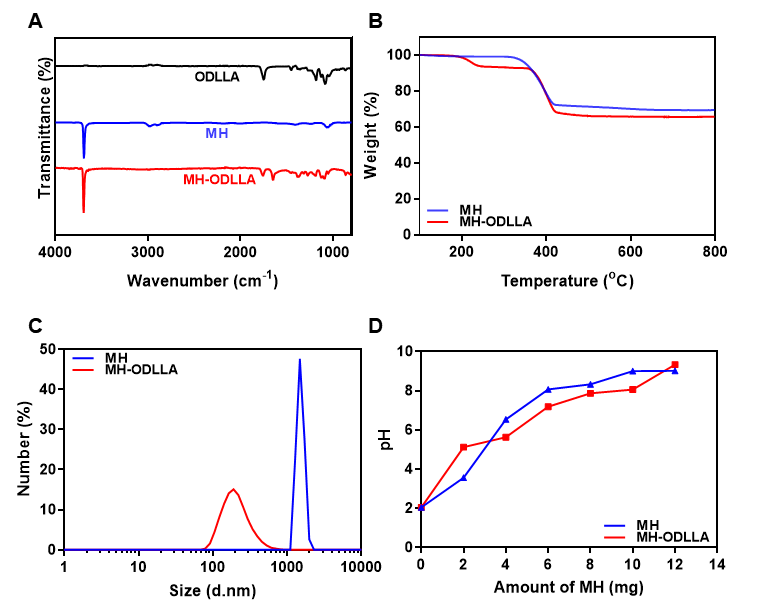
**

**Fig. S3.** **Characterization of modification MH (MH-ODLLA):** (A) FTIR spectra confirming the successful surface modification of magnesium hydroxide (MH) with oligo(D,L-lactide) (ODLLA), (B) thermogravimetric analysis (TGA) showing increased organic content in ODLLA-grafted MH (GF-MH), (C) particle size distribution analysis of MH and GF-MH, and (D) pH neutralization capacity demonstrating the buffering ability of GF-MH.

**
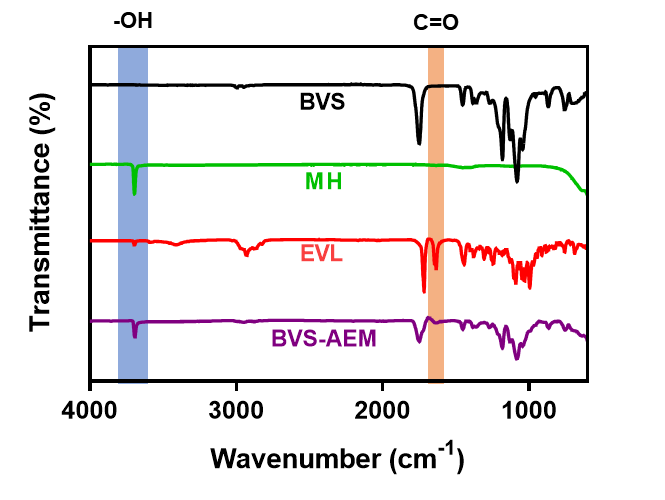
**

**Fig. S4.** ATR-FTIR spectra of unmodified BVS, MH, pure EVL, and BVS/AEM.

**Fig. S5.** Drug loading efficiency of MH in a coated BVS (BVS-AEMD).

**
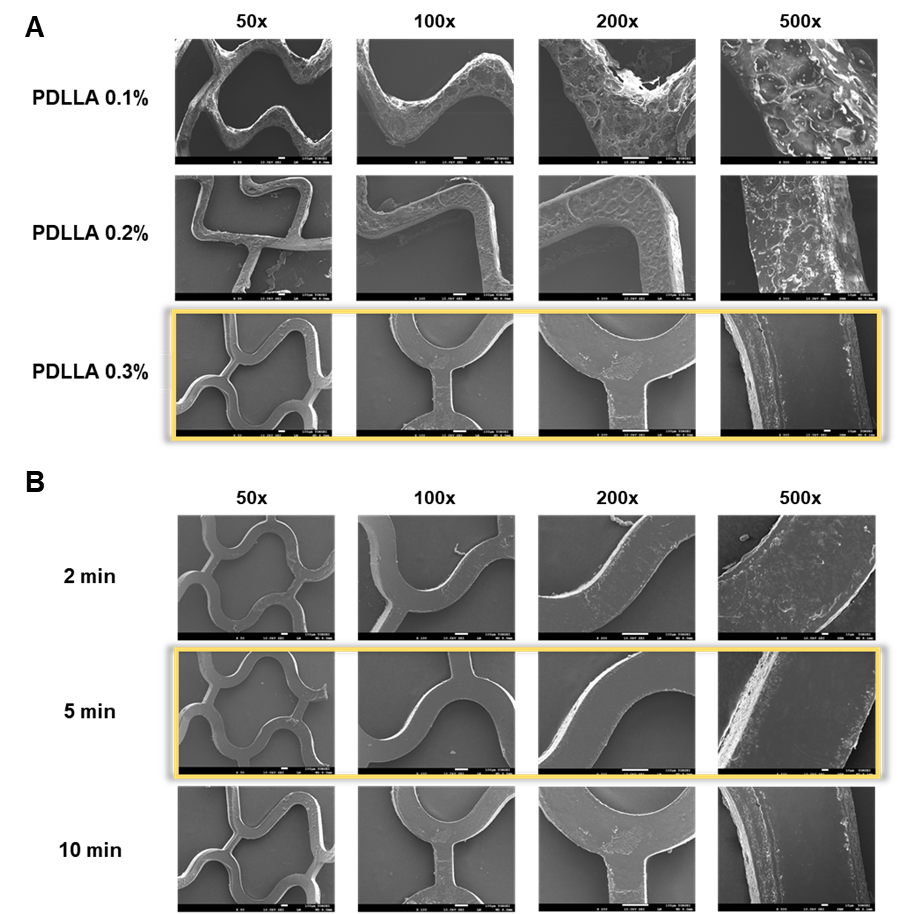
**

**Fig. S6.** Scanning electron microscopic images of the electrosprayed ALA-PDLLA particles on the luminal area of the BVS with variations of (a) PDLLA concentration and (b) spraying time. The scale bars represent 100 μm (in 50x, 100x, and 200x magnifications) and 10 μm (in 500x magnification).


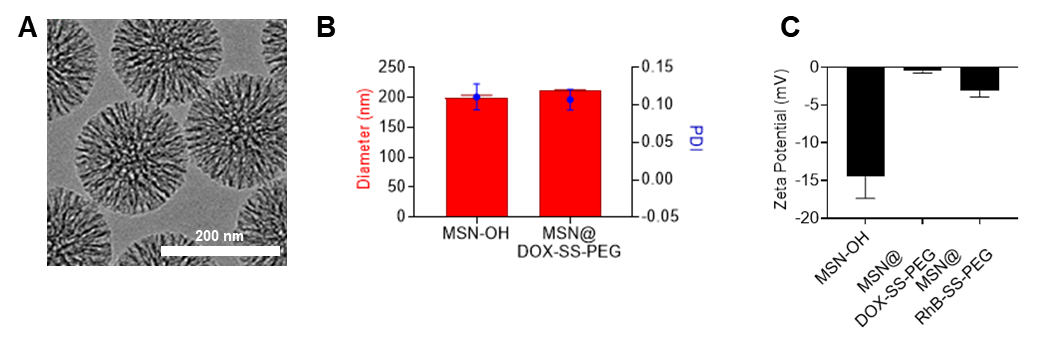


**Fig. S7.** **Characterization of dMSN:** (A) TEM image of dMSN-OH (Scale bar = 200 μm.), (B) Particle diameter and PDI of MSN-OH and MSN@DOX-SS-PEG, and (C) Zeta potential changes before and after PEGylation and DOX loading.

**Fig. S8.** Nitrogen adsorption–desorption isotherms of dMSN-SH showing specific surface area and pore size distribution.

**Fig. S9.** Release profile of Rhodamine B (RhB) from dMSN@RhB-SS-PEG–coated stent.

**
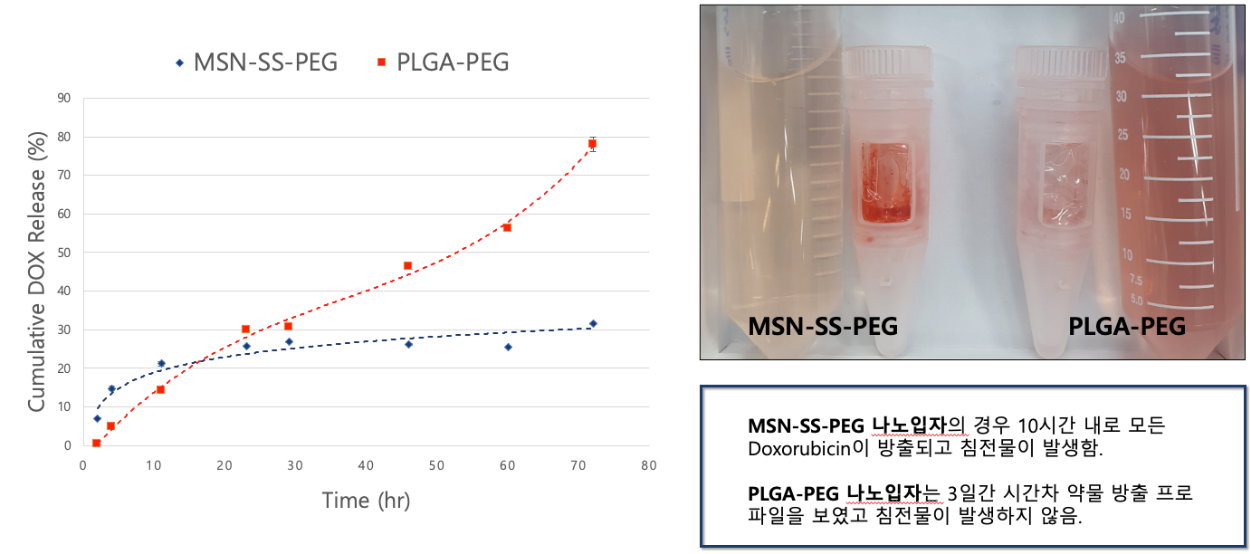
**

**Fig. S10.** Comparison of doxorubicin release profiles from MSN-SS-PEG and PEG-PLGA nanoparticles.


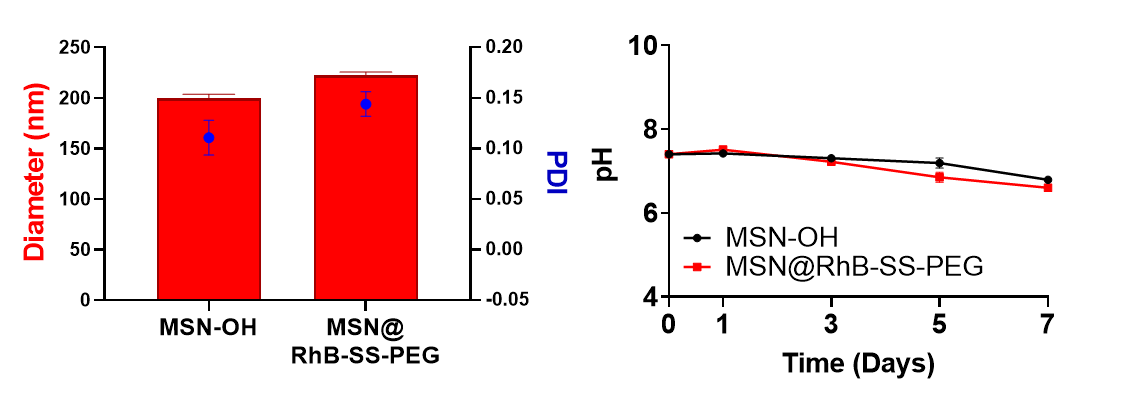


**Fig. S11.** Degradation behavior and pH variation of MSN-OH and MSN@RhB-SS-PEG nanoparticles over 7 days in PBS (pH 7.4) at 37 °C. Both samples maintained near-neutral pH, indicating stable degradation and minimal acidification during the incubation period.

**Fig. S12.** Cell viability at 12 h with various concentration of H_2_O_2_ treatment using HCAECs.


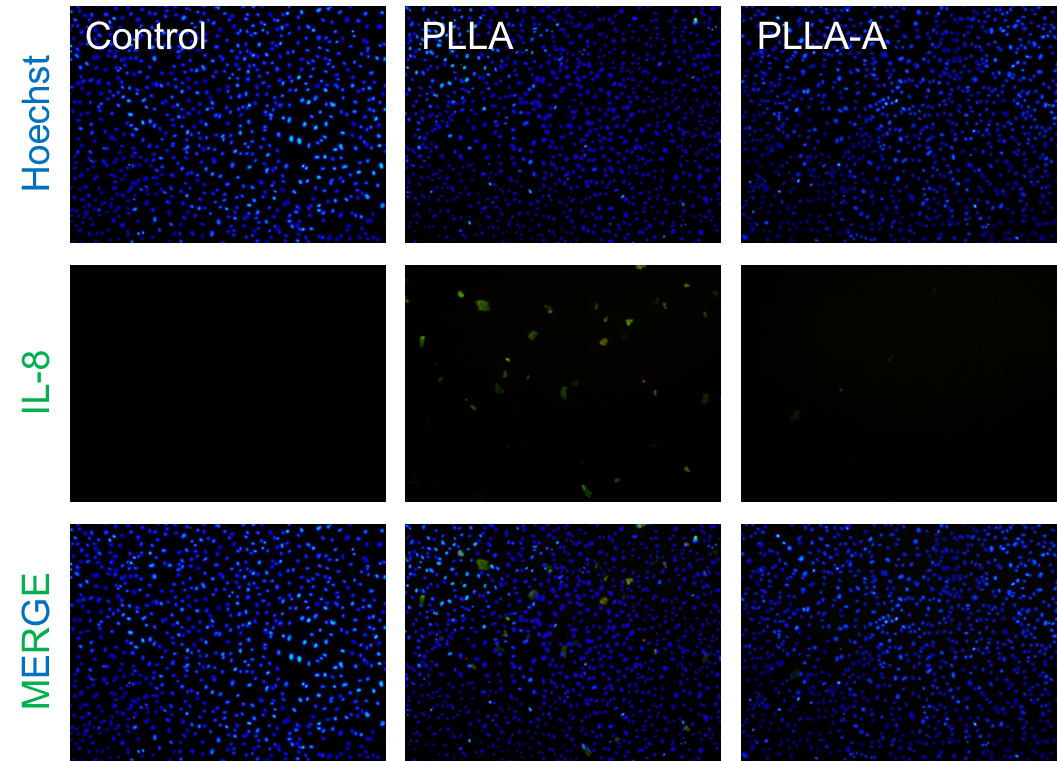


**Fig. S13.** Representative immunofluorescence images showing IL-8 expression in TNF-α-pretreated HCAEC cells incubated with scaffolds (Scale bar = 100 μm).
